# Introductory Review of Soft Implantable Bioelectronics Using Conductive and Functional Hydrogels and Hydrogel Nanocomposites

**DOI:** 10.3390/gels10100614

**Published:** 2024-09-25

**Authors:** San Kim, Yumin Shin, Jaewon Han, Hye Jin Kim, Sung-Hyuk Sunwoo

**Affiliations:** 1 Department of Chemical Engineering, Kumoh National Institute of Technology, Gumi 39177, Republic of Korea; 2 Division of Biomedical Engineering, Yonsei University, Wonju 26493, Republic of Korea; 3 Andrew and Peggy Cherng Department of Medical Engineering, California Institute of Technology, Pasadena, CA 91106, USA

**Keywords:** bioelectronics, conductive hydrogel, functional hydrogel, hydrogel nanocomposite, implantable electronics

## Abstract

Interfaces between implantable bioelectrodes and tissues provide critical insights into the biological and pathological conditions of targeted organs, aiding diagnosis and treatment. While conventional bioelectronics, made from rigid materials like metals and silicon, have been essential for recording signals and delivering electric stimulation, they face limitations due to the mechanical mismatch between rigid devices and soft tissues. Recently, focus has shifted toward soft conductive materials, such as conductive hydrogels and hydrogel nanocomposites, known for their tissue-like softness, biocompatibility, and potential for functionalization. This review introduces these materials and provides an overview of recent advances in soft hydrogel nanocomposites for implantable electronics. It covers material strategies for conductive hydrogels, including both intrinsically conductive hydrogels and hydrogel nanocomposites, and explores key functionalization techniques like biodegradation, bioadhesiveness, injectability, and self-healing. Practical applications of these materials in implantable electronics are also highlighted, showcasing their effectiveness in real-world scenarios. Finally, we discuss emerging technologies and future needs for chronically implantable bioelectronics, offering insights into the evolving landscape of this field.

## 1. Introduction

The functioning of many physiologically critical organs is primarily governed by electrical signals. The activation of electroactive organs, such as the brain [1,2], nerves [3,4], heart [5,6], and muscles [7,8], depends on ionic exchanges through their cellular membranes, which lead to changes in the ionic potential gradient [9,10]. Analyzing the location, amplitude, and pattern of these potential changes—referred to as electrophysiological signals—provides valuable insights into the state of these organs [11]. Moreover, artificial modulation of these electric potentials through external electrical stimulation can effectively control the activation and deactivation of electroactive organs. Given the critical importance of the bioelectrode-tissue interface for real-time monitoring and direct manipulation of these organs, wearable and implantable electronics are commonly employed for biointerfacing. Implantable bioelectronics, in particular, offer superior biointerfacing capabilities by establishing direct physical contact with target organs, free from interference by surrounding tissues.

**Figure 1:**
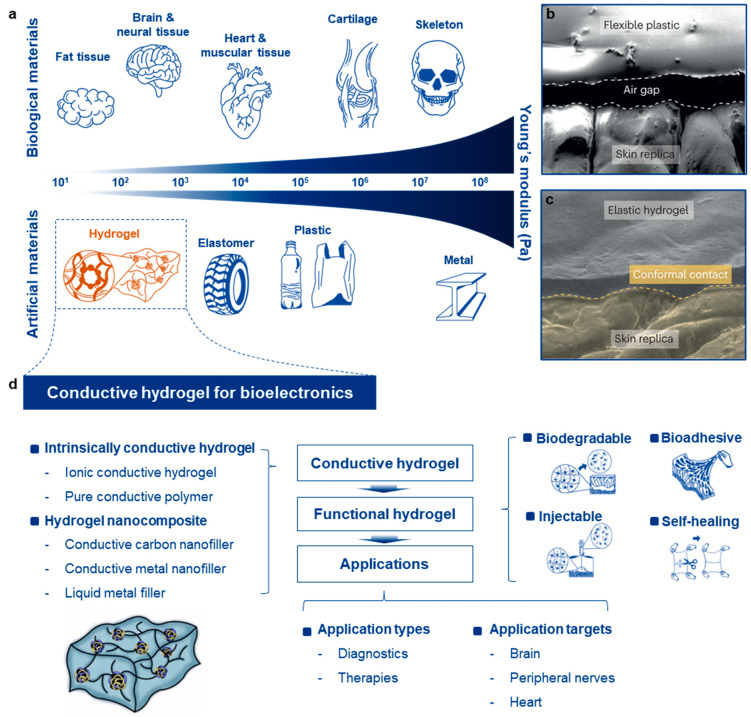
Overall structure of soft implantable bioelectronics using hydrogel and hydrogel nanocomposites. (**a**) Mechanical modulus of biological tissue and representative materials constituting bioelectronics. (**b**,**c**) Microscopic image showing conformal contact of flexible plastic (**b**) and soft hydrogel (**c**) on the skin surface. (**d**) Schematic illustration describing overall structure of the review.

Despite the advantages of implantable bioelectronics, conventional devices made from metal and silicon have been associated with various complications due to the mechanical mismatch between rigid electrodes and soft biological tissues (Figure 1a). First, rigid electrodes struggle to establish conformal contact with soft, curvilinear tissues, leading to the formation of an air gap that acts as an insulating layer, reducing the quality of biological signals and the efficiency of electrical stimulation (Figure 1b,c) [12]. Second, the rigid and sharp structure of these electrodes exacerbates implantation trauma in both acute and chronic phases. During implantation, the rigidity of the electrode can tear soft tissues, causing traumatic damage such as bleeding. In the chronic phase, the mechanical stress exerted by the rigid electronics on surrounding tissues in response to spontaneous macro- and micro-motions further reinforces inflammatory responses. The formation of thick fibrous scar tissue around the implant, which serves to isolate the device, further compromises the quality of the biointerface. Third, the high modulus of implantable electronics ironically increases the risk of device failure. Since implantable electronics are typically designed with smaller dimensions to reduce invasiveness, their rigidity, coupled with their thin structure, makes them brittle. This brittleness heightens the risk of device fracture both during and after implantation, leading to device failure and additional tissue damage. These complications highlight the critical need for intrinsically soft yet conductive materials to overcome these challenges.

Recently, there has been a significant surge of interest in soft and conductive nanocomposites as promising materials for implantable electronics. These soft nanocomposites consist of a physical blend of functional nanomaterials, which act as primary fillers, and elastomeric polymers, which form the matrix. The nanoscale fillers are evenly dispersed within the elastomeric matrix, creating a three-dimensional percolation network that enables the material’s electrical conductivity [13]. Importantly, the physical distribution of these nanosized fillers within the matrix allows for their relocation or rearrangement during mechanical deformation, preserving the integrity of the percolation network without significant loss of functionality. Different combinations of conductive fillers and elastomeric matrices yield nanocomposites with distinct properties. Among these, hydrogel-based nanocomposites have attracted particular attention due to their ultra-soft, tissue-like properties, high potential for functionalization, and water-rich composition, which enhances their suitability for effective biointerfacing.

This review aims to provide a concise overview of recent advancements in soft hydrogel nanocomposites for advanced implantable electronics, with an emphasis on making the topic accessible to general readers and those unfamiliar with the complexities of polymer science (Figure 1d). First, we will categorize the various materials that constitute hydrogel nanocomposites, introducing different types of hydrogels, including intrinsically conductive ones, and exploring their combinations with functional and conductive nanofillers. Next, we will examine the specific functionalities of hydrogel nanocomposites that are particularly advantageous for implantable electronics. These properties—such as biodegradability, tissue adhesiveness, injectability, and self-healing—can be achieved with hydrogels and offer significant benefits for implantable devices. We will then highlight recent studies that have applied hydrogel nanocomposites in implantable bioelectronics, showcasing their key roles in electrophysiological monitoring, electrical stimulation delivery, and interfacing with electroactive organs like the brain, peripheral nerves, heart, and other muscular tissues. Finally, we will address the current limitations of hydrogel nanocomposites and explore their future prospects, focusing on potential advancements and their contributions to the field of implantable electronics.

## 2. Conductive Hydrogels and Hydrogel Nanocomposites

Conductive hydrogels are advanced materials that combine the hydrophilic, soft properties of hydrogels with electrical conductivity, making them particularly valuable for biomedical applications [14] such as neural interfaces [15], tissue engineering [16], biosensors [17], and drug delivery systems [18]. These hydrogels can be classified into two main types based on their conductive mechanisms: intrinsically conductive hydrogels and hydrogels incorporating conductive nanofillers. Each type offers distinct advantages and employs different mechanisms to achieve conductivity. Conductive hydrogels, whether intrinsically conductive or nanocomposite-based, provide a versatile platform for a wide range of biomedical applications. The choice between these two types depends on specific application requirements, such as the desired level of conductivity, mechanical properties, and biocompatibility. Both types use unique mechanisms to achieve electrical conductivity, making them valuable for interfacing with biological tissues, promoting tissue regeneration, and enabling advanced biomedical devices. In this chapter, we will explore the conductive mechanisms of hydrogels and highlight representative examples of conductive hydrogels.

### 2.1. Intrinsically Conductive Hydrogels

Intrinsically conductive hydrogels are a unique class of materials that exhibit electrical conductivity without the need for added conductive fillers like nanoparticles or nanofibers. Instead, their conductivity is derived from the inherent properties of the polymer matrix, which can include the presence of conjugated bonds, ion exchange capabilities, and other intrinsic conductive mechanisms. In this section, we will explore these mechanisms in detail, focusing on how the polymer structure itself enables electrical conductivity. Understanding these intrinsic properties is key to leveraging the full potential of these hydrogels in biomedical applications, where conductivity, flexibility, and biocompatibility are crucial.

#### 2.1.1. Conjugated Polymer Backbone

Intrinsically conductive hydrogels often consist of polymers with conjugated systems, characterized by alternating single and double bonds along their backbone [19,20]. These conjugated systems form a network of π-electrons that can move freely along the polymer chain, facilitating electrical conductivity. In conjugated polymers, π-electrons are delocalized over the length of the polymer chain, creating a π-conjugated system that allows electrons to move along the backbone. The degree of delocalization depends on the length of the conjugated system and the molecular structure of the polymer. The mobility of these delocalized electrons significantly contributes to the material’s overall electrical conductivity. Greater conjugation and fewer defects in the polymer chain enhance conductivity, as extended conjugation and a regular polymer structure support better electron mobility.

**Figure 2:**
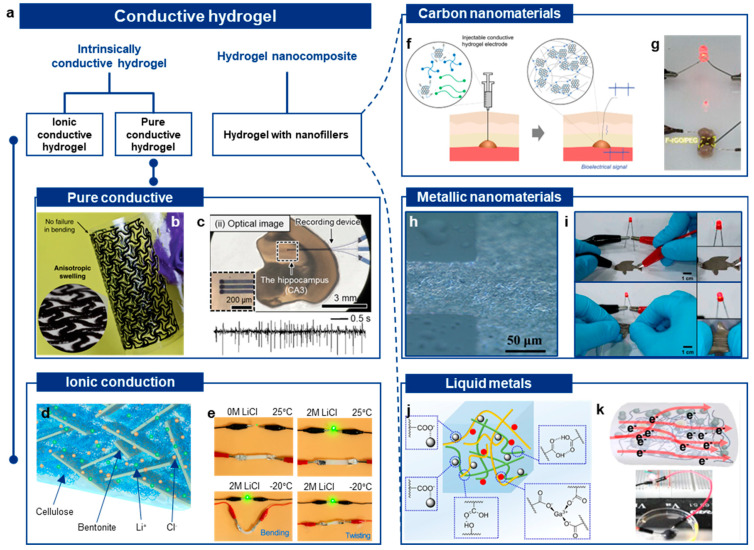
Conductive hydrogel and hydrogel nanocomposite. (**a**) Flowchart showing the conducting mechanism of hydrogel. (**b**,**c**) Pure conductive hydrogels. (**b**) Reproduced with permission from Ref. [31], Copyright 2019, Springer Nature. (**c**) Reproduced with permission from Ref. [32], Copyright 2022, AAAS. (**d**,**e**) Hydrogels with ionic conduction. Reproduced with permission from Ref. [33], Copyright 2022, Springer Nature. (**f**,**g**) Hydrogel nanocomposites with conductive carbon nanomaterials. Reproduced with permission from Ref. [34], Copyright 2023, John Wiley & Sons, Inc. (**h**,**i**) Hydrogel nanocomposites with conductive metallic nanofillers. (**h**) Reproduced with permission from Ref. [35], Copyright 2014, American Chemical Society. (**i**) Reproduced with permission from Ref. [36], Copyright 2019, AIP Publishing. (**j**,**k**) Conductive hydrogel nanocomposites containing liquid metals. (**j**) Reproduced with permission from Ref. [37], Copyright 2024, Elsevier. (**k**) Reproduced with permission from Ref. [38], Copyright 2021, Springer Nature.

Examples of intrinsically conductive polymers include polyaniline (PANI) [21,22], polypyrrole (PPy) [23,24], and poly(3,4-ethylenedioxythiophene):(styrene sulfonate) (PEDOT:PSS) [25,26,27,28]. These materials can form hydrogels with inherent conductivity due to their conjugated structures [29,30]. For instance, Lu et al. demonstrated a pure PEDOT:PSS hydrogel with remarkable electrical, mechanical, and swelling properties [31]. By adding dimethyl sulfoxide (DMSO) to an aqueous PEDOT:PSS solution, followed by dry annealing and rehydration, they achieved a hydrogel with superior electrical conductivity (~40 S/cm), a low Young’s modulus (~2 MPa), and high stretchability of over 35% strain (Figure 2b). In another example, Won et al. developed biocompatible, water-stable PEDOT:PSS hydrogels using laser-induced phase separation [32]. The laser delivered a strong electric field and photothermal energy to induce rapid phase separation of PEDOT:PSS. The resulting hydrogel exhibited high conductivity (670 S/cm), Young’s modulus of 57 MPa, and an elongation of 20%. This PEDOT:PSS hydrogel was successfully implanted in the brain and sciatic nerve of animals, where it recorded neural signals and delivered neural stimulation (Figure 2c).

Doping is a process in which the polymer is chemically modified to introduce charge carriers, either electrons or holes, thereby enhancing its conductivity [39,40]. In intrinsically conductive hydrogels, doping can be achieved by incorporating dopants either during or after polymer synthesis. P-doping involves adding electron acceptors (oxidizing agents) that remove electrons from the polymer, creating holes (positive charge carriers) [41]. This increases the concentration of holes, thus enhancing the conductivity. For instance, doping polyaniline (PANI) with protonic acids increases its conductivity by converting the emeraldine base (non-conductive form) into the emeraldine salt (conductive form) [42,43].

On the other hand, N-doping involves the introduction of electron donors (reducing agents) that add electrons to the polymer, increasing the number of free electrons (negative charge carriers) [39,44,45]. Although N-doping is less commonly used in conductive hydrogels, it can be applied in specialized applications. The degree of doping, which is controlled by the concentration of dopants, directly influences the level of conductivity. Generally, higher doping levels result in greater conductivity, but they may also impact the stability and mechanical properties of the hydrogel. Thus, finding a balance between doping concentration and material performance is crucial for optimizing the properties of intrinsically conductive hydrogels.

#### 2.1.2. Ionic Conductive Hydrogel

In addition to electronic conductivity, intrinsically conductive hydrogels can also exhibit ionic conductivity, which involves the movement of ions through the hydrated polymer matrix [46,47]. This type of conductivity is particularly relevant in biological environments, where ionic conduction plays a critical role. The hydrogel matrix, being highly hydrated, enables the dissociation of ionic species, allowing ions to move freely within the water-rich environment of the hydrogel, thereby contributing to ionic conductivity. Some conductive hydrogels contain functional groups that can exchange ions with the surrounding medium. For example, sulfonate groups in sulfonated polystyrene or carboxyl groups in polyacrylic acid can facilitate the exchange of cations, such as H⁺ [48,49], Na⁺ [50,51], and K⁺ [52,53,54], with the environment. In certain cases, ionic conductivity can work in tandem with electronic conductivity, particularly in redox-active polymers. The movement of ions can induce changes in the oxidation state of the polymer, which in turn affects its overall conductivity. Ionic conductivity is especially important for applications like biosensors, where the hydrogel’s response to changes in ionic concentration can be used for sensing, and in bioelectronics, where the material interfaces with biological tissues that naturally conduct ions. This dual conductivity—electronic and ionic—makes intrinsically conductive hydrogels versatile materials for a wide range of biomedical applications.

Proton conduction is a specific form of ionic conductivity in which protons (H⁺) act as the charge carriers [55,56]. This mechanism is especially significant in proton-conducting polymers and can greatly enhance the overall conductivity of a hydrogel. Protons move through the hydrogel matrix via a hopping mechanism, in which they transfer from one hydrogen-bonded water molecule or functional group to another. This process relies on the presence of a continuous hydrogen-bond network, often supported by water molecules or proton-specific functional groups like sulfonic acid (-SO_3_H) [57,58,59]. Additionally, protons can move within hydration shells surrounding ions or polar groups, further enhancing their mobility.

Polymers with sulfonic acid groups (e.g., sulfonated polystyrene) or phosphonic acid groups are commonly used for proton conduction [60,61,62]. These materials can form hydrogels with high proton conductivity, making them useful in applications such as fuel cells and biosensors. For example, Wang et al. developed a cellulose hydrogel with a robust bond network, focusing on enhancing the mechanical strength without increasing the crosslinking density [33]. They incorporated bentonite nanoplatelets, which exhibit a high elastic modulus, to strengthen the hydrogel and create straight channels for rapid ion transport (Figure 2d). When LiCl was introduced, the hydrogel achieved a tensile stress of 0.76 MPa, a fracture strain of 96%, and ionic conductivity of 89.9 mS/cm. LiCl also inhibited the solidification of the hydrogel, enabling high electrical conductivity even at low temperatures. The resulting anti-freezing hydrogel could power a light-emitting diode at temperatures as low as −20 °C, and continued to function even when bent or twisted (Figure 2e).

#### 2.1.3. Redox-Active Hydrogels

Some intrinsically conductive hydrogels are also redox-active, meaning they can undergo reversible oxidation and reduction reactions [63,64]. This property enhances conductivity through redox coupling, where electron transfer reactions are linked with ionic movements. The presence of redox-active centers within the polymer matrix enables charge transfer processes, as these centers can exchange electrons with their surroundings, contributing to the material’s overall conductivity. In redox-active hydrogels, changes in the polymer’s oxidation state can lead to the simultaneous movement of both ions and electrons, further improving conductivity [65,66]. This combination of redox activity and conductivity makes these hydrogels particularly valuable for applications like bioelectronic interfaces and electrochemical sensors, where they can facilitate electron transfer between biological systems and electronic devices. Redox-active conductive hydrogels thus serve as an effective platform for interfacing with biological tissues, enhancing sensor performance, and advancing bioelectronics.

### 2.2. Hydrogel Nanocomposites

Conductive hydrogel nanocomposites are advanced materials that incorporate conductive nanofillers into a hydrogel matrix, resulting in a composite with enhanced electrical conductivity, mechanical strength, and other functional properties. These nanocomposites can be fabricated either by mixing nanofillers into the hydrogel precursor or by forming the hydrogel around a pre-existing freestanding nanofiller network [67]. This combination allows the hydrogel nanocomposite to simultaneously exhibit the exceptional electrical conductivity of the conductive fillers and the mechanical softness and biocompatibility of the hydrogel matrix. As a result, these materials can effectively address the limitations of conventional materials, such as bulk metals, silicon, and pure hydrogels, which often face challenges related to mechanical mismatch and limited flexibility. By leveraging the complementary properties of both the hydrogel matrix and the nanofillers, these nanocomposites are highly suitable for a wide range of applications, including wearable and implantable sensors and stimulators [68,69], drug delivery systems [70,71], and soft robotics [72,73]. These attributes make conductive hydrogel nanocomposites a promising platform for next-generation biomedical and soft electronic technologies.

The percolation threshold refers to the minimum concentration of nanofillers required to establish a continuous conductive network within the hydrogel matrix [74,75,76]. Below this threshold, the composite behaves as an insulator, but once the threshold is exceeded, significant conductivity emerges. At high nanofiller concentrations beyond the percolation threshold, the nanofillers form a percolative network that enables efficient electron transport through the hydrogel matrix [77,78]. The connectivity and uniform distribution of the nanofillers are crucial for achieving high conductivity, as they ensure the formation of a continuous conductive pathway. At lower concentrations, where the nanofillers do not form a complete network, quantum tunneling can allow electrons to transfer between closely spaced fillers, particularly at short distances. This tunneling effect still contributes to the overall conductivity of the composite. The hydrogel’s high water content further enhances conductivity by facilitating the movement of ions, which is especially important in biological environments where ionic conduction plays a critical role. Additionally, the interface between the hydrogel matrix and the nanofillers can significantly impact charge transfer and distribution. Good interfacial compatibility ensures efficient electron transport across the matrix and nanofillers while maintaining the mechanical integrity of the composite. This interplay between the conductive nanofillers, hydrogel matrix, and interface is key to optimizing the performance of conductive hydrogel nanocomposites for applications in bioelectronics and soft materials.

#### 2.2.1. Constituting Materials

The hydrogel matrix is a three-dimensional network of hydrophilic polymers that can retain a significant amount of water, providing both structural support and biocompatibility for the composite. Common polymers used for the matrix material include poly(vinyl alcohol) (PVA), poly(ethylene glycol) (PEG), polyacrylamide (PAAm), alginate, chitosan, and gelatin. These polymers are selected based on their ability to form hydrogels with desirable mechanical properties, such as flexibility, elasticity, and durability, as well as their compatibility with conductive nanofillers. The combination of these polymers with nanofillers ensures the hydrogel matrix can maintain its mechanical integrity while supporting the conductive network, making it suitable for various biomedical and soft electronic applications.

Nanofillers are materials with at least one dimension in the nanoscale range, and they are incorporated into the hydrogel matrix to enhance electrical conductivity and other functional properties. Conductive nanofillers can be broadly categorized into two types: carbon-based nanomaterials and metallic nanomaterials, depending on their primary composition.

Carbon-based nanofillers include graphene, carbon nanotubes (CNTs), and carbon fibers [79,80]. Graphene is a single layer of carbon atoms arranged in a hexagonal lattice [81,82,83], typically fabricated through exfoliation from graphite. It is known for its exceptional electrical conductivity, high surface area, and mechanical strength. Graphene oxide, a derivative of graphene containing oxygen-functional groups, is water-dispersible and compatible with hydrogels [84,85,86]. CNTs are another widely used carbon-based nanomaterial, consisting of a graphene sheet rolled into a cylindrical structure, though methods like arc discharge, laser ablation, and chemical vapor deposition are more commonly employed for their fabrication. CNTs offer excellent electrical conductivity, mechanical strength, and thermal stability, and can be classified as either single-walled (SWCNTs) or multi-walled (MWCNTs) [87,88,89]. Carbon fibers are elongated carbon structures with high aspect ratios, providing good electrical conductivity and mechanical properties.

For example, Park et al. developed an injectable conductive hydrogel composite by mixing poly(ethylene glycol)-tetrathiol and poly(ethylene glycol)-diacrylate with reduced graphene oxide (Figure 2f) [34]. The resulting hydrogel demonstrated remarkable properties, including softness (16.2 kPa), electroconductivity (22.02 mS/cm), biodegradability, and injectability. Even when applied to a curved surface, the conductive hydrogel was capable of powering light-emitting diode (LED) bulbs (Figure 2g). Additionally, Park et al. showed that the hydrogel has great potential as a bioelectrode for recording electromyogram signals in rat skin, highlighting its effectiveness in bioelectronic applications.

Thanks to their intrinsic metallic conductivity and nanoscale geometry, metallic nanomaterials can be dispersed within a hydrogel matrix to impart electrical conductivity [90,91]. These nanomaterials—such as nanoparticles, nanowires, and nanorods—offer high conductivity due to their metallic nature [92]. The fabrication of these nanomaterials generally follows one of two approaches: top-down or bottom-up. In the top-down approach, bulk metals are etched or reduced into smaller particles, while the bottom-up process involves the aggregation of metallic atoms into a lattice structure, where metallic ions are chemically reduced to form the nanomaterial [93].

Zero-dimensional metal nanoparticles, such as gold (Au) [94,95], silver (Ag) [96,97], copper (Cu) [98,99], and platinum (Pt) nanoparticles [100,101], offer excellent electrical conductivity along with additional functionalities like catalytic activity or antimicrobial properties. One-dimensional metal nanowires, including silver nanowires (AgNWs) [35,102], gold nanowires (AuNWs) [103,104], and copper nanowires (CuNWs) [105], have high aspect ratios that allow them to form percolative networks, significantly enhancing conductivity [12,106]. Metal nanorods, which are cylindrical in shape, provide a balance between the high conductivity of nanowires and the dispersibility of nanoparticles [107,108].

For instance, Ahn et al. developed highly conductive AgNW-based microelectrodes using a photolithographic process, which allowed the electrodes to be transferred onto a hydrogel substrate (Figure 2h) [35]. The AgNW-based hydrogel electrode exhibited high electrical conductivity (109 Ω) and mechanical flexibility, enduring 100 bending cycles. In another example, Lim et al. blended AgNWs into a tough alginate hydrogel, achieving both high conductivity and stretchability [36]. The AgNW-hydrogel nanocomposite demonstrated remarkable conductivity (9.1 Ω) and maintained its electrical properties even with 30% stretchability (Figure 2i). This hydrogel was successfully applied in various wearable electronics, including wireless communication antennas and supercapacitors, highlighting its potential in flexible, wearable technologies.

Recently, liquid metals have gained attention as filler materials due to their high conductivity and fluidic properties [109,110]. These materials include metals or metal alloys that are in a liquid state at or near room temperature. Commonly used liquid metals include gallium (Ga), gallium-based alloys, and indium-tin alloys. Gallium has a melting point of approximately 29.8 °C, making it liquid just above room temperature. Gallium-based alloys, such as eutectic gallium-indium (EGaIn), which melts at around 15.5 °C, are popular due to their low toxicity and excellent conductivity [111]. Indium-tin alloys, when combined with gallium (e.g., Galinstan), form low-melting-point alloys with favorable properties for use in hydrogels.

Thanks to their liquid-like properties, liquid metals can form a percolation network within the porous hydrogel matrix, enabling efficient conductivity [112,113]. For example, Zhou et al. developed a sodium alginate hydrogel that stabilized liquid metal nanoparticles in a colloidal suspension (Figure 2j) [37]. The alginate formed a microgel around the liquid metal nanoparticles, encapsulating them to provide colloidal stability and ensure interfacial compatibility between the liquid metal nanoparticles and the poly(acrylic acid) matrix. The resulting hydrogel nanocomposite exhibited high stretchability, self-healing, self-adhesiveness, and high sensitivity.

In another example, Xu et al. proposed a strategy for the convergent synthesis of a biomimetic electroconductive hydrogel nanocomposite comprising liquid metal [38]. The hydrogel nanocomposite was self-assembled by mixing polysaccharides, conductive biopolymers (PEDOT), and liquid metal microdroplets in the presence of tannic acid. The resulting hydrogel demonstrated electrical conductivity (~0.05 S/cm), adhesive strength, and high biocompatibility in both in vitro and in vivo studies (Figure 2k). These advances highlight the potential of liquid metal-based hydrogels for applications requiring stretchability, conductivity, and biocompatibility.

Recently, strategies have emerged to combine metallic nanomaterials with ionic conductive hydrogels, merging the benefits of both ionic conductivity and hydrogel nanocomposites. For example, Wang and Daoud reported a conductive hydrogel with a hybrid conductive mechanism that integrates ionic conductivity from metal ions (Ag⁺ and Cu^2^⁺) with silver nanowires embedded in a crosslinked chitosan matrix [114]. This hybrid hydrogel demonstrated high conductivity and excellent durability, making it suitable for use as a motion/temperature sensor, while also incorporating an energy harvesting module. This approach capitalizes on both the ionic and electronic conduction pathways, enhancing the performance and functionality of the hydrogel for advanced applications in wearable electronics and sensing devices.

#### 2.2.2. Synthesis Methods

There are several synthesis methods for hydrogel nanocomposites. In situ polymerization involves the polymerization of monomers in the presence of dispersed nanofillers within the hydrogel matrix. This method ensures uniform distribution of nanofillers and strong interactions between the matrix and nanofillers. In solution blending methods, the nanofillers are dispersed in a solution of the hydrogel-forming polymer, followed by gelation through processes such as cooling, pH change, or chemical crosslinking. The layer-by-layer assembly method involves the sequential deposition of alternate layers of the hydrogel matrix and nanofillers, allowing precise control over the composite structure. Finally, the electrostatic assembly method uses electrostatic interactions to assemble charged nanofillers and polymers into a composite structure.

## 3. Functional Hydrogel and Hydrogel Nanocomposites for Advanced Biointerfacing

Although the mechanical softness, water-rich nature, and overall improved biocompatibility of hydrogels make them favorable for biomedical applications, their potential for functionalization further enhances their appeal. Functional properties such as biodegradability, bioadhesiveness, injectability, and self-healing equip hydrogels for a wide range of in vivo applications. In this section, we will introduce the mechanisms behind these functional properties in hydrogels and hydrogel nanocomposites, along with representative examples of their biomedical applications.

### 3.1. Biodegradable Hydrogels and Hydrogel Nanocomposites

#### 3.1.1. Degradation Mechanisms

Biodegradable hydrogels naturally degrade into water-soluble monomers or sub-components within the body [115,116,117]. The degradation process varies depending on the materials used to form the hydrogels. Common hydrogels made from natural polymers like gelatin, chitosan, and alginate degrade through hydrolysis, a chemical process where water molecules cleave the polymer chains [118,119]. Hydrolysis typically occurs in hydrogels with ester or amide bonds. For instance, poly(lactic-co-glycolic acid) (PLGA) hydrogels undergo hydrolysis of ester bonds, releasing lactic and glycolic acids, which are naturally metabolized [120,121].

Hydrogels can also degrade through enzymatic cleavage, where specific enzymes, such as proteases, catalyze the breakdown of peptide sequences or polymer structures [122,123]. The susceptibility of a hydrogel to enzymatic degradation depends on its chemical structure. Oxidative degradation occurs when hydrogels are exposed to reactive oxygen species (ROS), leading to the breakdown of polymer chains, particularly in hydrogels with unsaturated bonds. For example, hydrogels containing oxidizable moieties like PEG with pendant unsaturated groups can undergo oxidative degradation in the presence of ROS [124,125].

Physical erosion refers to the dissolution or disintegration of hydrogels caused by water or bodily fluids, influenced by factors such as crosslinking density and water-soluble components. For example, PVA-based hydrogels may erode when exposed to water [126,127]. Some hydrogels are also broken down by cells through processes such as phagocytosis, where cells actively internalize and degrade the hydrogel [128,129]. Hydrogels containing components that cells can metabolize can promote tissue integration, eventually replacing the hydrogel with native tissue.

**Figure 3:**
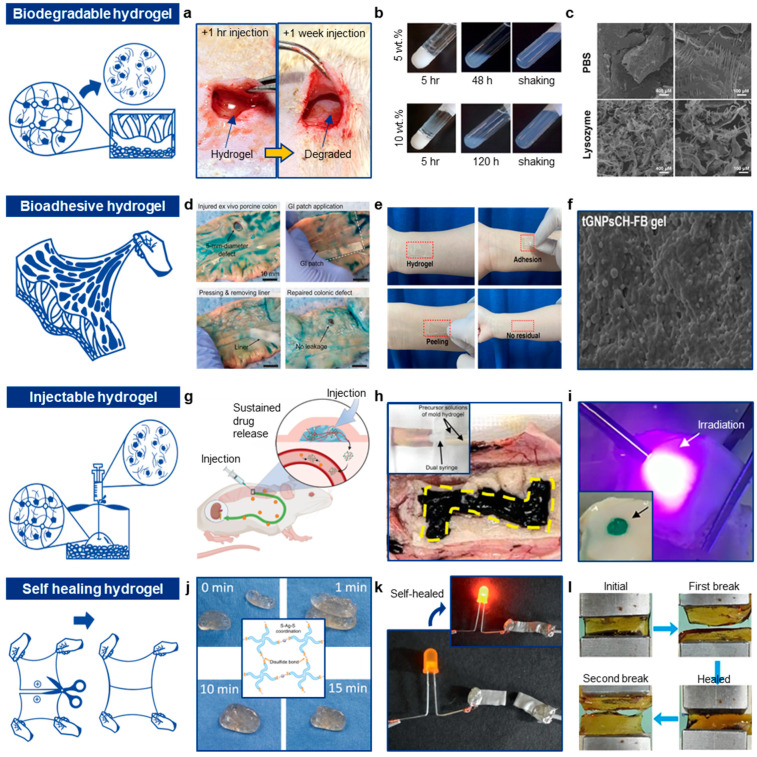
Advanced properties of soft implantable hydrogels. (**a**–**c**) Biodegradable hydrogels. (**a**) Reproduced with permission from Ref. [130], Copyright 2022, American Chemical Society. (**b**) Reproduced with permission from Ref. [131], Copyright 2018, American Chemical Society. (**c**) Reproduced with permission from Ref. [132], Copyright 2024, American Chemical Society. (**d**–**f**) Tissue-adhesive hydrogels. (**d**) Reproduced with permission from Ref. [133], Copyright 2022, AAAS. (**e**) Reproduced with permission from Ref. [134], Copyright 2023, Elsevier. (**f**) Reproduced with permission from Ref. [135], Copyright 2018, American Chemical Society. (**g**–**i**) Injectable hydrogels. (**g**) Reproduced with permission from Ref. [136], Copyright 2022, John Wiley & Sons, Inc. (**h**) Reproduced with permission from Ref. [137], Copyright 2023, John Wiley & Sons, Inc. (**i**) Reproduced with permission from Ref. [138], Copyright 2021, AAAS. (**j**–**l**) Self-healing hydrogels. (**j**) Reproduced with permission from Ref. [139], Copyright 2017, Springer Nature. (**k**) Reproduced with permission from Ref. [140], Copyright 2023, Springer Nature. (**l**) Reproduced with permission from Ref. [139], Copyright 2017, Springer Nature.

Understanding these degradation mechanisms is essential for tailoring hydrogels to specific biomedical applications. Researchers can control the rate and extent of biodegradation by adjusting factors such as polymer composition, crosslinking density, and incorporating specific chemical functionalities. For instance, Cha et al. developed a multifunctional injectable hydrogel with properties such as stretchability, bioadhesion, and biodegradability [130]. Two separate hydrogel precursor solutions were used: (i) one containing oxidized hyaluronic acid and tyramine with calcium ions, and (ii) the other containing carboxymethyl chitosan, horseradish peroxidase, and hydrogen peroxide, both of which were injected via a dual syringe. Upon injection, multiple spontaneous reactions—such as click reactions, enzymatic reactions, and ionic interactions—enabled rapid gelation. Over time, the injected hydrogel underwent degradation via hydrolysis. Cha et al. demonstrated that the injected hydrogel was fully dissolved within 7 days after injection (Figure 3a).

#### 3.1.2. Constituting Hydrogels

There are numerous biodegradable hydrogels that degrade spontaneously in the body through the mechanisms mentioned earlier. Gelatin hydrogels, derived from collagen, a natural protein found in animal tissues [141,142], exhibit tunable elasticity based on concentration and crosslinking density. Gelatin hydrogels primarily degrade through enzymatic processes, with enzymes such as collagenases breaking down the peptide chains of gelatin [143,144,145]. Hyaluronic acid hydrogels, widely used in biomedical applications, are composed of a natural polysaccharide found in the extracellular matrix [146,147]. These hydrogels are degraded enzymatically by hyaluronidases, which break down hyaluronic acid into smaller fragments [148,149]. Combining gelatin and hyaluronic acid to form gelatin-hyaluronic acid hydrogels creates materials with tunable elasticity, making them suitable for applications requiring mechanical flexibility [150]. These hydrogels also degrade enzymatically [151]. Chitosan hydrogels, derived from chitin, a polysaccharide found in the exoskeletons of crustaceans [152,153], can be modified for varying mechanical strengths depending on the degree of deacetylation and crosslinking. Chitosan hydrogels degrade through enzymatic hydrolysis by chitosanases, lysozymes, and other enzymes [154,155]. Additionally, chemical hydrolysis can break down chitosan hydrogels. Alginate hydrogels, derived from alginic acid, commonly obtained from brown seaweed [156,157], exhibit mechanical strength influenced by the presence of divalent cations such as Ca^2^⁺ [158]. These hydrogels degrade through hydrolysis of glycosidic linkages and enzymatic mechanisms, resulting in the slow release of alginic acid. Dextran hydrogels, composed of polysaccharides with glucose units [159,160], are enzymatically degraded by dextranases and other glycosidases [161].

Synthetic polymers such as PEG hydrogels [162], PVA hydrogels [163], polylactic acid (PLA) hydrogels [164], polyglycolic acid (PGA) hydrogels, and poly(lactic-co-glycolic acid) (PLGA) hydrogels also exhibit good biodegradability. PEG hydrogels, with repeating ethylene glycol units, undergo hydrolysis to break ester or ether linkages [165]. PVA hydrogels degrade through physical erosion and hydrolysis of acetate groups [166]. PLA hydrogels degrade through the hydrolysis of ester linkages, producing lactic acid, which is metabolized by the body [167]. Similarly, the ester linkages in PGA and PLGA hydrogels hydrolyze, yielding glycolic acid and a combination of lactic and glycolic acids, respectively.

These hydrogels exhibit diverse biodegradation mechanisms based on their chemical compositions and structures, allowing for tailored degradation profiles in various biomedical applications. For example, Pertici et al. reported a symmetric pentablock copolymer platform consisting of PLA, PEG, and PNIPAAm (PNIPAAm_60_-*b*-PLA_14_-*b*-PEG_91_-*b*-PLA_14_-*b*-PNIPAAm_60_) [131]. The amphiphilic structure of this copolymer allows gel formation through intermicellar crosslinking upon heating. With the hydrolysis of the PLA block, the pentablock copolymer degrades into lower molar mass polymer chains (Figure 3b). This platform enables the delivery of hydrophobic drugs loaded into the micelle core to specific regions, such as the rat brain striatum, a target often affected by neurological disorders. In an in vitro trial, the riluzole (50 μg) drug-loaded hydrogel showed degradation kinetics similar to the non-loaded hydrogel. In another example, Liang et al. developed an injectable hydrogel composed of carboxymethyl chitosan and bioadhesive nanoparticles based on polylactic acid-hyperbranched polyglycol [132]. This injectable hydrogel maintained its activity and released drugs in a sustained manner over 7 days. The hydrogel’s biodegradability was assessed by incubation with lysozyme. Accelerated degradation was confirmed by SEM images, which showed that the hydrogel structure was significantly disrupted after lysozyme addition (Figure 3c).

The biodegradation of conductive hydrogels requires careful design, as materials that contribute to conductivity, such as silver ions, can be cytotoxic upon degradation. To address this challenge, researchers have focused on developing fully biodegradable conductive hydrogels made exclusively from biocompatible materials. For instance, Hardman et al. demonstrated a versatile ionic gelatin/glycerol hydrogel that offers high stability, biocompatibility, and complete biodegradability [168]. This hydrogel is also capable of functioning as a strain sensor, withstanding strains of up to 454%. This innovative design shows the potential for creating conductive hydrogels that maintain both functionality and safety in biomedical applications.

### 3.2. Bioadhesive Hydrogels and Hydrogel Nanocomposites

Bioadhesiveness is another highly favorable characteristic of hydrogels and hydrogel nanocomposites for biomedical applications. Bioadhesive hydrogels have the ability to adhere to biological tissues upon contact, forming strong bonds with tissue surfaces. This property is essential for various biomedical applications, including wound closure, tissue repair, drug delivery, and the attachment of biomedical devices.

A notable recent example is the work by Choi et al., who developed a bioelectronic cardiac patch capable of adhering to wet cardiac surfaces [169]. This system achieved conformable tissue adhesion in less than one second without requiring external stimuli. The researchers employed a mussel-inspired adhesive polymer, known for its exceptional wet adhesion properties and film-like characteristics. The hydrogel patch showed great potential for chronic applications on cardiac surfaces, making it suitable for long-term monitoring of cardiac activity. The bioadhesive mechanisms of bioadhesive hydrogels can be classified into six categories: physical interactions, chemical interactions, electrostatic interactions, molecular recognition, mucosal adhesion, and mechanical interactions. Many bioadhesive hydrogels achieve strong adhesion to tissues by employing two or more of these adhesion strategies simultaneously, enhancing their effectiveness for various biomedical applications.

#### 3.2.1. Physical Interactions

The first bioadhesive mechanism involves physical interactions, such as hydrogen bonding and Van der Waals forces. Hydrogels containing functional groups like hydroxyl (-OH), carboxyl (-COOH), ester (-COO-), or amine (-NH_2_) can form hydrogen bonds with functional groups on tissue surfaces, promoting adhesion. For example, chitosan-based hydrogels contain amino groups that can form hydrogen bonds with hydroxyl or carboxyl groups on tissue surfaces [170]. Similarly, the hydroxyl groups in hydroxypropyl methylcellulose (HPMC) hydrogels can bond with hydroxyl groups on tissue glycoproteins and mucins, while hydroxyl groups in PVA hydrogels can form hydrogen bonds with tissue surfaces [171]. Catechol groups can also engage in hydrogen bonding and metal ion coordination, further enhancing adhesion [172].

Van der Waals forces, as non-covalent interactions, can occur between hydrogels and tissue surfaces, further strengthening adhesion. For instance, Wu et al. developed a patch for sealing gastrointestinal (GI) organs [133]. The patch’s bottom layer, made from PAA-NHS/PVA hydrogel, adhered to injured GI tissue by forming hydrogen bonds with the tissue surface. The fully swollen GI patch exhibited high interfacial toughness (>350 J/m^2^ for colon, >500 J/m^2^ for stomach) and excellent biocompatibility (Figure 3d). In another example, Wang et al. fabricated an adhesive PAA/PVA-B hydrogel for wipe sampling [134]. The adhesive force in this hydrogel was enhanced by both hydrogen bonds and Van der Waals forces, resulting in a peel strength of over 3.48 N/m on various substrates, including human tissue and metals (Figure 3e).

Additionally, Jiang et al. developed a skin adhesive hydrogel patch using a protein-polyphenol complexation strategy [173]. This hydrogel exhibited an adhesion energy of approximately 8.1 J/m^2^ to porcine skin. Notably, the hydrogel adhered well at human body temperature but lost its adhesive properties below 10 °C, demonstrating temperature-sensitive adhesion.

#### 3.2.2. Chemical Interactions

The second bioadhesive mechanism involves chemical interactions, where some bioadhesive hydrogels contain reactive groups that form covalent bonds with functional groups on tissue surfaces. For instance, hydrogels with reactive groups such as acrylates or epoxides can form covalent bonds with tissue proteins. Covalent bonds can be classified into two types: reversible and irreversible.

Amide bonds are the most commonly used static (irreversible) covalent bonds due to their high stability, strength, and biocompatibility. These static bonds are often utilized in vivo when long-term adhesion is required [174]. Conversely, dynamic bonds offer the advantage of being detachable on demand. For example, gelatin-methacryloyl (GelMA) hydrogels, formed by modifying gelatin with methacryloyl groups, enable UV- or visible light-induced crosslinking. During crosslinking, GelMA forms covalent bonds with tissue proteins, while gelatin itself can form hydrogen bonds with these proteins [175]. PEG-based hydrogels can be functionalized with adhesive peptides, such as RGD peptides, which specifically bind to integrin receptors on cell surfaces. Additionally, fibrin hydrogels, derived from the blood protein fibrinogen, form covalent bonds through the action of thrombin, which converts fibrinogen to fibrin, enabling adhesion to tissues (Figure 3f) [135]. Some hydrogels functionalized with catechol groups (e.g., dopamine-modified polymers) can form strong covalent bonds with amine or thiol groups on tissue surfaces. Liang et al. developed a dual-dynamic-bond crosslinked antibacterial adhesive hydrogel [176], incorporating dynamic catechol-Fe bonds and Schiff base bonds, which allows for reversible adhesion. Additionally, some hydrogels contain components that crosslink with tissue proteins to form strong bonds; for example, hydrogels with aldehyde groups can react with amine groups in tissue proteins, forming covalent bonds.

#### 3.2.3. Electrostatic Interactions

The third bioadhesive mechanism involves electrostatic interactions, which are relatively strong but can be weakened by the presence of water in tissues, reducing stability. Hydrogels containing charged groups, such as carboxylate (-COO⁻), ammonium (-NH_3_⁺), or sulfonic acid (-SO_3_H), can interact with oppositely charged groups on tissue surfaces, thereby enhancing adhesion. For example, alginate, a natural polysaccharide derived from brown algae, forms hydrogels in the presence of divalent cations like calcium ions (Ca^2^⁺), which create stable ionic bonds with the tissue’s extracellular matrix. The alginate hydrogel can also swell and intermix with tissue structures, further improving adhesion [177]. Similarly, the positively charged amino groups in gelatin can interact electrostatically with negatively charged groups on tissues, such as sulfates and carboxylates. For instance, Tian et al. improved the stability of electrostatic adhesion by adding water-absorbent gelatin and 2-methoxyethyl acrylate, which contains hydrophobic groups, to enhance adhesion in wet environments [178]. In another example, Huang et al. developed hybrid hydrophobic hydrogels by incorporating sodium tripolyphosphate (STPP) [179]. These hydrogels exhibited reduced hydration levels and increased strength due to electrostatic interactions between the hydrogel components, resulting in strong adhesion to various substrates.

#### 3.2.4. Mechanical Interactions

Some hydrogels adhere to tissues through swelling and intermingling. Upon contact with tissue surfaces, these hydrogels can swell, allowing them to intermingle with tissue structures and form physical entanglements that enhance adhesion. For instance, hydroxypropyl methylcellulose (HPMC) hydrogels hydrate and swell upon contact with tissues, forming mechanical contact with the tissue surface [180]. Similarly, PEG hydrogels can swell, creating a hydrated matrix that adheres to tissues [181]. In addition to swelling, the hydroxyl groups on HPMC can form hydrogen bonds with hydroxyl groups on tissue glycoproteins and mucins.

The fibrin hydrogel network can also intermingle with tissue structures, providing physical adhesion through entanglement. This type of adhesion, involving swelling and intermingling, is particularly useful in applications like microneedle fixation. For example, Yang et al. designed microneedles with swellable hydrogel tips [182]. The hydrogel’s molecular structure can be tailored to conform to the topography of the tissue surface, ensuring close contact and enhanced adhesion.

#### 3.2.5. Molecular Recognitions and Mucosal Applications

Through the mechanism of molecular recognition, bioadhesive hydrogels can be engineered with ligands that specifically bind to receptors or molecules on tissue surfaces, thereby enhancing adhesion. For example, Fan et al. developed a bioadhesive hydrogel using chitosan and poly(acrylic acid-co-catechol) [183]. This hydrogel can form various reversible non-covalent bonds with other substances and exhibits a 180-degree peeling adhesion strength of approximately 1010 J/m^2^ on porcine skin. Similarly, Zhao et al. fabricated physical double-network hydrogel adhesives [184], incorporating receptors that enable the hydrogel to form specific bonds with tissue surfaces.

Bioadhesive hydrogels designed for mucosal applications can adhere to mucosal tissues through mechanisms such as hydration, hydrogen bonding, and electrostatic interactions with mucin molecules. For instance, Xu et al. developed a catechol-modified chitosan hydrogel [185], which demonstrated enhanced mucoadhesion in vitro for over 6 h. In another example, Vakili et al. improved the mucoadhesion of cellulose nanocrystals by incorporating polyacrylic acid [186].

By leveraging these adhesive mechanisms, bioadhesive hydrogels can achieve strong and durable adhesion to biological tissues, offering a versatile platform for various biomedical applications. The selection of adhesive mechanisms depends on the specific requirements of the application and the characteristics of the target tissue.

### 3.3. Injectable Hydrogels

Injectability is a crucial property for hydrogels used in biomedical applications, referring to the ability of hydrogels to be delivered through a syringe or catheter into the body in a minimally invasive manner. Typically, injectable hydrogels are administered as fluidic precursors through a needle or catheter [187]. Upon exposure to external stimuli, such as temperature, pH, or ionic strength, these precursors undergo crosslinking reactions and sol-gel transitions [188]. This process allows the hydrogel to be delivered into the body through small openings, such as those created by a needle, regardless of the final shape or volume of the gel [189]. Injectable hydrogels are versatile and can be applied in various biomedical fields. For instance, Shukla et al. developed an injectable hydrogel composed of drug-loaded brush copolymers in methyl cellulose, designed for controlled drug release over an extended period [190]. This injected hydrogel demonstrated significant melanoma suppression without causing adverse side effects, in contrast to conventional chemotherapy, which often results in severe toxicity.

Furthermore, injectable hydrogels offer several advantages, including minimal invasiveness, improved biocompatibility, enhanced tissue adhesiveness, and on-demand gelation. For instance, these hydrogels can be delivered through extremely fine needles, eliminating the need for invasive and burdensome open surgery. This reduces trauma associated with surgical procedures, minimizes the risk of infection and immune response, and promotes faster recovery. Additionally, before undergoing the sol-gel transition, injectable hydrogels exhibit high softness, allowing them to closely conform to complex, curvilinear structures within the body, thus forming a conformal contact.

Another advantage is on-demand gelation, where the gelation process can be controlled by adjusting external stimuli such as temperature, light, or pH. This provides flexibility in timing the gelation, which is particularly useful in clinical settings. In this section, we will explore the various mechanisms of injectability in hydrogels and present representative examples.

#### 3.3.1. Thermosensitive Injectable Hydrogels

Thermosensitive injectable hydrogels are a class of hydrogels that undergo a reversible sol-gel phase transition in response to temperature changes [191]. These hydrogels are typically in a liquid state at lower temperatures and form a gel at physiological temperatures (around 37 °C) [192]. This property makes them particularly useful for minimally invasive applications, as they can be easily injected in their liquid form and then solidify upon reaching body temperature, conforming to the shape of the target site.

Thermosensitive hydrogels are usually made from polymers that exhibit a lower critical solution temperature (LCST) or an upper critical solution temperature (UCST). LCST polymers are soluble in water below a certain temperature (LCST) but become insoluble and form a gel above this temperature [193]. A common example is poly(N-isopropylacrylamide) (PNIPAAm), which has an LCST of around 32 °C [194,195]. On the other hand, UCST polymers gel below a certain temperature (UCST) and dissolve above it [196], but LCST polymers are more commonly used in biomedical applications.

Block copolymers, such as Pluronic (also known as Poloxamer), are widely used in thermosensitive hydrogels [197]. Pluronic F127, a triblock copolymer consisting of a central hydrophobic polypropylene oxide (PPO) block flanked by two hydrophilic polyethylene oxide (PEO) blocks [198,199], is a well-known example. This structure allows the polymer to form micelles in solution, which aggregate and form a gel at higher temperatures. The balance between hydrophilic and hydrophobic interactions in the polymer chains is crucial to the sol-gel transition. As the temperature increases, hydrophobic interactions dominate, causing polymer aggregation and gel formation.

At lower temperatures, the polymer chains are hydrated and remain in a liquid or sol form, with hydrophilic interactions dominating. As the temperature approaches the LCST, the polymer undergoes a conformational change [200] where the hydrophobic segments interact more strongly with each other, leading to the formation of micelles or aggregated structures. Once the temperature exceeds the LCST, further aggregation occurs, resulting in the formation of a three-dimensional network [201]. This network traps water molecules and other components, leading to gelation, driven by increased hydrophobic interactions and reduced solvation at higher temperatures.

Thermosensitive hydrogels are used for sustained and controlled drug delivery [202]. Drugs can be encapsulated within the gel matrix and released slowly as the hydrogel degrades or via diffusion [203]. These hydrogels also serve as scaffolds for cell delivery and tissue regeneration, providing a supportive environment for cell growth and designed to degrade as new tissue forms. Additionally, thermosensitive hydrogels can be applied to wounds in a liquid form, then solidify into a protective gel barrier at body temperature. This barrier helps keep the wound moist and can deliver therapeutic agents, such as growth factors or antibiotics.

For instance, Park et al. demonstrated an injectable hydrogel by integrating crosslinked hyaluronic acid into Pluronic F127 for extended deferoxamine release [136]. This hydrogel exhibited thermosensitive rheological properties and provided sustained release of deferoxamine, an iron nanochelator used in chelation therapy (Figure 3g). The sustained release (up to 14 days) of deferoxamine nanoparticle nanochelators reduced the discomfort of repeated subcutaneous injections of deferoxamine nanoparticles.

#### 3.3.2. pH-Sensitive Gelation

pH-sensitive injectable hydrogels are a type of smart hydrogel that undergoes a sol-gel transition in response to changes in pH [204]. These hydrogels are typically liquid within a specific pH range and form a gel when exposed to a different pH environment, usually corresponding to the physiological conditions of the target tissue [205]. This property makes them ideal for applications requiring localized treatment, as gelation can be triggered by the pH of the surrounding tissue.

pH-sensitive hydrogels are composed of polymers that contain ionizable groups [206], such as carboxyl [207], amine [208], and sulfonate groups [209], which can accept or donate protons in response to pH changes. Anionic polymers, which contain acidic groups (e.g., carboxyl groups), ionize in basic conditions and are neutral in acidic environments, while cationic polymers, containing basic groups (e.g., amine groups), ionize in acidic conditions and are neutral in basic environments. Examples of anionic polymers include natural polymers like alginate [210] and synthetic ones like poly(acrylic acid) (PAA) [211] and poly(methacrylic acid) (PMAA) [212]. Cationic polymers include natural polymers such as chitosan [213] and synthetic polymers like poly(2-dimethylaminoethyl methacrylate) (PDMAEMA) [214].

pH-sensitive hydrogels may form physical or chemical crosslinks. Physical crosslinking often involves ionic interactions between ionizable groups, while chemical crosslinking forms covalent bonds through chemical reactions. In the sol state, the hydrogel precursors are liquid, with ionizable groups either fully ionized or neutral, depending on the solution’s pH. This allows for easy injection through a needle or catheter. For anionic polymers, the carboxylic groups become protonated in lower pH environments [211], reducing electrostatic repulsion between polymer chains and inducing gelation through increased hydrophobic interactions or hydrogen bond formation. For cationic polymers, amine groups can be deprotonated at higher pH, similarly reducing electrostatic repulsion and promoting gelation [215].

Upon reaching the target site with the appropriate pH, the hydrogel transitions from liquid to gel. The transition is influenced by factors such as the degree of ionization, polymer concentration, and the presence of crosslinkers. Some pH-sensitive hydrogels can reversibly transition between sol and gel states based on pH changes, which can be beneficial for applications like controlled drug release. For example, Qu et al. presented injectable anti-bacterial conductive hydrogels for drug delivery, which respond to both electric fields and pH changes [216]. The hydrogel, composed of a chitosan-graft-polyaniline copolymer and a dextran crosslinker, exhibited controlled degradability, making it an intelligent carrier for drug delivery.

#### 3.3.3. Ion-Induced Gelation

Ion-induced injectable hydrogels are a type of smart hydrogel that undergo gelation in response to the presence of specific ions, typically divalent cations such as calcium (Ca^2^⁺) [217] or magnesium (Mg^2^⁺) [218]. These hydrogels are especially useful in biomedical applications requiring controlled and localized gelation [219]. The mechanism of ion-induced gelation involves interactions between the ionizable groups on the polymer and the ions in the surrounding environment, leading to the formation of a crosslinked network.

Ion-induced hydrogels are commonly made from polymers with ionizable groups that can interact with cations. The most frequently used polymers include alginate [219], chitosan [220], and hyaluronic acid [221]. Alginate, a natural polysaccharide derived from brown seaweed, consists of blocks of mannuronic acid (M) and guluronic acid (G) residues, with carboxyl groups that interact with divalent cations. Chitosan can be crosslinked with multivalent anions or negatively charged molecules, while hyaluronic acid can form hydrogels in the presence of divalent cations. Divalent cations such as calcium (Ca^2^⁺), magnesium (Mg^2^⁺), and barium (Ba^2^⁺) [222] are commonly used to induce gelation, forming ionic crosslinks between polymer chains. The ionizable groups (e.g., carboxyl groups in alginate) are essential for the gelation process, as they interact with crosslinking ions to form a stable network.

In the sol state, hydrogel precursors are in liquid form, allowing easy injection. The polymers are dissolved in an aqueous solution without crosslinking ions, maintaining low viscosity and high mobility. After injection, the introduction of divalent cations initiates gelation. This can occur naturally within the body if the injected solution contacts ion-rich tissues, or gelation can be facilitated by co-injecting the ion source.

For example, in alginate, calcium ions form ionic crosslinks with carboxylate groups on the guluronic acid residues, leading to the formation of an “egg-box” structure. This structure provides mechanical stability, transforming the liquid solution into a gel. The ionic crosslinking results in a three-dimensional network that traps water and other components, creating a stable hydrogel. The mechanical properties and stability of the gel can be controlled by adjusting the concentrations of the polymer and the crosslinking ions.

#### 3.3.4. Enzymatic Crosslinking

Enzymatic crosslinking of injectable hydrogels is a process in which enzymes catalyze the formation of covalent bonds between polymer chains, resulting in hydrogel gelation [223]. This method is highly specific, occurs under mild conditions, and offers fine control, making it ideal for biomedical applications. Enzymatic crosslinking is often used for in situ gelation, where hydrogel precursors are injected in a liquid form and solidify into a gel upon exposure to specific enzymes present in the body or co-injected with the hydrogel [224].

The polymers used in enzymatic crosslinking contain functional groups that are targeted by enzymes. These include natural polymers like fibrinogen [225], gelatin [226], and silk fibroin [227], which can be crosslinked by enzymes such as thrombin [228] or transglutaminase [229]. Additionally, synthetic polymers containing enzyme-sensitive groups, such as lysine [230] or glutamine residues [231], can also be crosslinked by transglutaminase. Enzymes, as biological catalysts, facilitate covalent bond formation between specific functional groups on polymer chains. For example, thrombin catalyzes the conversion of fibrinogen to fibrin, forming a fibrin gel, while transglutaminase catalyzes covalent bond formation between lysine and glutamine residues on proteins or synthetic peptides. Other enzymes like laccase [232] and peroxidase [233] can crosslink phenolic or other oxidizable groups on polymers.

In the sol state, hydrogel precursors—comprising a polymer solution and an enzyme or substrate—are in a liquid form, allowing them to be easily injected through a syringe or catheter. The enzyme specifically recognizes and binds to its substrate on the polymer chains, ensuring that crosslinking occurs only at intended sites, minimizing unwanted reactions. As the enzyme catalyzes covalent bond formation, a three-dimensional polymer network forms, trapping water and other components, leading to gelation. After injection, the gelation occurs in situ, creating a solid or semi-solid gel at the target site. The gel’s properties, such as stiffness and porosity, depend on the degree of crosslinking and the concentrations of both enzyme and polymer [234].

For example, Cha et al. demonstrated injectable hydrogels that can be patterned using a minimally invasive method [137]. Two individual hydrogel precursors, containing a crosslinking mediator (Ca^2^⁺ ion) and an enzyme solution, were injected via a dual syringe. During injection, the precursor solutions mixed, enabling enzymatic crosslinking. After gelation, the hydrogel was physically carved using a needle or catheter to create a mold. The frame hydrogel precursor solution was then injected into the vacant space of the carved mold hydrogel. Following ionic gelation of the frame gel, an enzyme solution was injected to degrade the mold gel, leaving the patterned frame hydrogel behind (Figure 3h). By incorporating iron nanoparticles into the frame gel, wireless thermal activation was successfully demonstrated.

#### 3.3.5. Photocrosslinking Hydrogel

Photocrosslinking is a versatile technique for forming hydrogels by utilizing light to initiate chemical reactions that crosslink polymer chains, creating a three-dimensional network [235]. This method is particularly advantageous for injectable hydrogels as it allows precise spatial and temporal control over the gelation process. Photocrosslinkable hydrogels are highly valuable in biomedical applications such as drug delivery, tissue engineering, and wound healing [236].

Photocrosslinkable hydrogels are typically made from polymers containing photo-reactive groups [237]. These polymers can be derived from natural, synthetic, or hybrid materials. For example, natural polymers such as gelatin methacryloyl (GelMA) [238] and hyaluronic acid methacrylate (HAMA) [239] can be photocrosslinked after being modified with photo-reactive groups like methacryloyl. Synthetic polymers like poly(ethylene glycol) diacrylate (PEGDA) [240] and poly(ethylene glycol) dimethacrylate (PEGDMA) [241] can also be functionalized with these groups. Photo-reactive groups, such as acrylates, methacrylates, and vinyl groups, form covalent bonds upon exposure to light, initiating polymerization and crosslinking to form networks.

Photo-initiators are compounds that absorb light and generate reactive species, such as free radicals, to initiate polymerization. The choice of photo-initiator depends on the wavelength of light used for crosslinking. Common photo-initiators include Irgacure 2959 for UV light and lithium phenyl-2,4,6-trimethylbenzoylphosphinate for visible light [242,243].

In the sol state, hydrogel precursors, consisting of a polymer solution and photo-initiator, are in liquid form, allowing easy injection through a syringe or catheter. Upon exposure to light (UV or visible) [244], the photo-initiator absorbs the energy and generates reactive species, typically free radicals. These radicals initiate the polymerization of photo-reactive groups (e.g., acrylates or methacrylates) on the polymer chains, which crosslink to form a stable network. The gel’s properties, such as mechanical strength and porosity, can be fine-tuned by adjusting exposure time, light intensity, and the concentration of photo-reactive groups and initiator.

For example, Hua et al. developed a versatile photocrosslinkable hydrogel by combining photoinitiated radical polymerization and photoinduced imine crosslinking to form double-network hydrogels [138]. This hydrogel was created using methacrylate-modified hyaluronic acid for photoinitiated radical polymerization and o-nitrobenzyl-grafted hyaluronic acid and gelatin for photoinduced imine crosslinking. The hydrogel gelled rapidly, within 1.2 s, upon light exposure (Figure 3i). This photocrosslinkable hydrogel was applied as an autologous chondrocyte scaffold for cartilage repair in a swine model with articular cartilage defects.

### 3.4. Self-Healing Hydrogel

An implantable self-healing hydrogel is a type of hydrogel designed for placement inside the body, either temporarily or permanently, for various biomedical applications. The key characteristic of these hydrogels is their ability to autonomously repair structural damage [245,246]. This self-healing capability is facilitated by dynamic covalent bonds, non-covalent interactions, or other reversible mechanisms that allow the hydrogel network to reform after disruption [247]. These mechanisms enable the hydrogel to restore its original structure and properties after mechanical damage, such as cracking, tearing, or other forms of physical disruption.

Implantable electronic devices are increasingly being integrated with hydrogel materials due to the unique properties of hydrogels, such as biocompatibility, flexibility, and high water content. In the context of implantable electronics, self-healing hydrogels offer several significant advantages [248]. First, they can maintain electrical conductivity and prolong the device’s lifespan [249]. For electronic applications, maintaining conductivity is critical for the device’s functionality. Damage to the hydrogel, such as cracks or tears, can disrupt conductive pathways and cause device failure. Self-healing hydrogels can autonomously repair these disruptions, restoring electrical pathways and ensuring continuous operation. This ability to repair minor damage can extend the operational lifespan of the device, reducing the need for replacement surgeries and minimizing associated risks.

Second, self-healing hydrogels can maintain biocompatibility and safety. Biocompatibility is essential for any material implanted in the body to prevent adverse immune responses and ensure patient safety. Damage to the hydrogel could expose non-biocompatible components or cause mechanical irritation. Self-healing properties help maintain the hydrogel’s barrier function and biocompatibility, preventing the exposure of potentially harmful materials and ensuring a stable interface with biological tissues.

Finally, self-healing hydrogels ensure functional integration with biological tissue. Implantable electronic devices often interact with biological tissues, such as in neural interfaces, biosensors, or drug delivery systems. Maintaining stable and functional integration with surrounding tissues is crucial for accurate monitoring and treatment. Self-healing hydrogels can adapt to dynamic biological environments, recovering from deformation or damage caused by bodily movements and maintaining consistent interactions with tissues.

#### Self-Healing Mechanisms

The self-healing mechanisms in implantable hydrogels are based on their ability to autonomously repair damage and restore mechanical and functional properties. These mechanisms involve various chemical and physical processes occurring at the molecular or supramolecular level.

Dynamic covalent bonds are reversible covalent bonds that can break and reform under specific conditions, such as changes in pH, temperature, or the presence of a catalyst [250]. These bonds allow the hydrogel network to self-heal after being damaged [251,252]. For instance, imine bonds, formed by the condensation reaction between an aldehyde (or ketone) and an amine, can break in acidic conditions and reform in neutral or basic conditions [253,254,255], enabling reversible reformation and self-healing. Disulfide bonds, formed by the oxidation of thiol groups, can be reduced back to thiols and reoxidized, allowing for bond reformation [256]. For example, Chen et al. developed an injectable self-healing hydrogel with antibacterial and angiogenic properties for diabetic wound regeneration [139]. The hydrogel, synthesized by crosslinking thiolated polyethylene glycol with silver nitrate to form -S-Ag-S- bonds, could self-heal within 10 min after being halved (Figure 3j). The gradual degradation of the hydrogel also released antibacterial silver ions for sustained antibacterial effects. Boronic ester bonds, formed between boronic acid and diols, can reversibly dissociate and reform in response to pH changes or competing diols [257,258,259]. Hydrazone bonds, formed by the reaction of hydrazides with aldehydes or ketones [260,261], can undergo reversible hydrolysis, enabling self-healing.

Supramolecular interactions involve non-covalent bonds such as hydrogen bonding [262], ionic interactions [263,264], and van der Waals forces [265,266,267]. These interactions are inherently reversible, making them suitable for self-healing applications. Hydrogen bonding occurs between a hydrogen donor and an acceptor, allowing hydrogels with multiple hydrogen-bonding groups to re-establish these bonds after damage [268,269]. For example, Zhao et al. developed a self-healing conductive organogel composite made of poly(vinyl alcohol)-sodium borate (PVA-borax) gel with a percolating network of silver microflakes and a gallium-based liquid metal alloy [140]. The composite exhibited high electrical conductivity (7 × 10^4^ S/m), low Young’s modulus (~20 kPa), and high stretchability (>400% strain). The hydrogen bonds between PVA and borax could easily re-form after cutting, enabling mechanical self-healing, while the liquid metal facilitated electrical self-healing (Figure 3k).

Ionic interactions between oppositely charged groups [270,271] allow hydrogels containing polyelectrolytes to heal by dissociating and reforming ionic crosslinks [139,272]. π-π stacking, involving interactions between aromatic rings [273,274], facilitates self-healing by allowing reorganization of aromatic units. For instance, Han et al. developed a mussel-inspired polydopamine-polyacrylamide hydrogel with exceptional toughness, high self-healing ability, cell affinity, and tissue adhesiveness [139]. This hydrogel, capable of withstanding strains up to 3300%, demonstrated remarkable toughness (fracture energy of 2400 J/m^2^, higher than human cartilage’s ~1000 J/m^2^). After rupture, the hydrogel fully self-healed without external stimuli, thanks to reversible non-covalent interactions like hydrogen bonds and π-π stacking. In tensile-heal-tensile tests, the hydrogel withstood large strains after 2 h of healing at room temperature (Figure 3l).

Host-guest chemistry involves inclusion complex formation between a host molecule (e.g., cyclodextrin) and a guest molecule, which can dissociate and reform, enabling self-healing [275,276]. Hydrophobic interactions between non-polar groups in an aqueous environment [277,278] can drive the self-assembly of hydrophobic domains, which reorganize after damage. In hydrogels containing amphiphilic molecules, hydrophobic segments form micelles in water [279,280]. After damage, these micelles reassemble, contributing to self-healing. Hydrogels with block copolymers exhibit phase separation, with hydrophobic domains clustering together. When disrupted, these domains reassemble after stress is released, leading to self-healing.

Physical reorganization refers to the movement and realignment of polymer chains or domains within the hydrogel to repair damage. Polymers with flexible chains can move and diffuse to close gaps or cracks, driven by thermal energy or external stimuli [281,282]. In hydrogels with semi-permanent network structures, the relaxation of the network over time can allow gradual healing of mechanical defects [283].

Enzyme-mediated self-healing uses enzymes to catalyze bond formation or cleavage, enabling self-healing [284,285]. For instance, enzymes like transglutaminase can form covalent bonds between polymer chains. If these bonds break, the enzyme can reform them, facilitating self-healing. Dynamic covalent bonds can also be cleaved and reformed by enzymes, enabling targeted self-healing in biological environments.

## 4. Functional Conductive Hydrogels for Monitoring Biological Signals and Therapeutic Applications 

Conductive hydrogels with diverse functionalities exhibit high biocompatibility due to their mechanical properties being closely aligned with those of biological tissues [286]. Their hydrophilic, three-dimensional network structure not only provides excellent mechanical properties but also facilitates superior liquid exchange and transport capabilities [287]. These features enable conductive hydrogels to adhere seamlessly to the surface of skin or organs, minimizing mechanical mismatch. Their low surface impedance allows for accurate measurement and effective treatment of physiological electrical signals [288].

This section explores the various applications of conductive hydrogels as biological interfaces. In particular, it examines how conductive hydrogels can be used to monitor physiological electrical signals in the brain, heart, and other organs effectively. Additionally, it highlights their potential for disease treatment, neural and tissue regeneration, and wound healing.

### 4.1. Monitoring of Physiological Electrical Signals

Conductive hydrogels possess both electrical signal transmission capabilities and biocompatibility, making them highly effective biological interfaces within the microenvironment of biological tissues. As a result, these hydrogels are capable of consistently and accurately monitoring various physiological signals, such as electroencephalography (EEG), electrocorticography (ECoG), electrocardiography (ECG), and electromyography (EMG), over extended periods of time. This prolonged monitoring capability allows for comprehensive assessments of physiological conditions, thereby improving the effectiveness of both disease monitoring and therapeutic interventions.

#### 4.1.1. Brain Activity Monitoring

**Figure 4:**
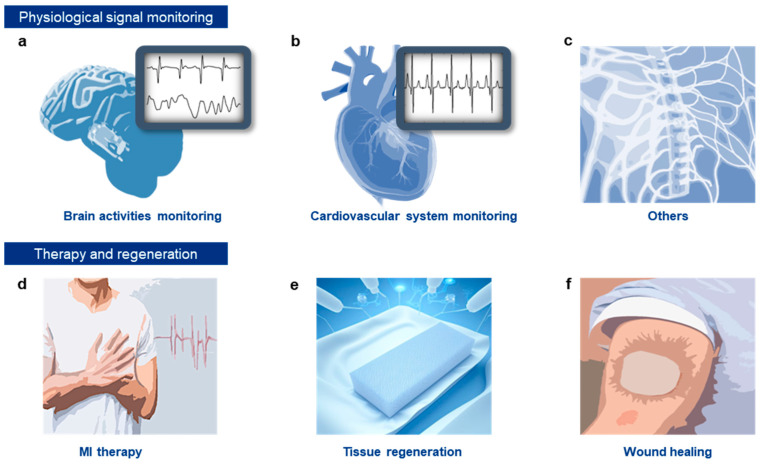
Functional conductive hydrogels for monitoring and therapeutic applications, classified into: (**a**–**c**) Monitoring biological signals; (**d**–**f**) therapeutic applications. (**a**) Brain activities monitoring. (**b**) Cardiovascular system monitoring. (**c**) Other types of biological signal monitoring. (**d**) MI therapy. (**e**) Tissue regeneration. (**f**) Wound healing.

EEG and ECoG signals are essential for reflecting the brain’s electrical activity and are critical tools for evaluating brain function (Figure 4a) [289]. EEG signals, which are recorded non-invasively from the scalp at single or multiple sites, exhibit slow rhythms (5–300 µV, <100 Hz). However, non-invasive scalp recordings often lack sufficient accuracy due to signal attenuation [10]. To achieve more precise measurements, direct electrode implantation is frequently required for monitoring EEG signals [290]. In contrast, ECoG signals, characterized by intermediate rhythms (0.01–5 mV, <200 Hz), can be monitored using electrodes directly affixed to the brain’s surface.

For effective brain electrical activity measurement, electrodes must be fabricated from flexible materials that minimize disturbance during implantation [291]. Given the brain’s low Young’s modulus (~4 kPa), hydrogels with comparable mechanical properties are particularly well-suited for brain interfacing [292]. The flexibility of conductive hydrogels reduces damage caused by brain micro-movements and pulsations, minimizing immune responses and preventing fibrosis at the electrode interface. Consequently, conductive hydrogel-based biointerfaces provide stable and consistent signal acquisition over extended periods, making them ideal materials for monitoring brain electrical activity.

#### 4.1.2. ECG Monitoring

Cardiovascular diseases are a leading cause of global mortality, according to the World Health Organization [293]. Prevention of these diseases often relies on electrocardiogram (ECG) monitoring, a well-established method known for its effectiveness in diagnosing cardiac conditions [97,294]. Since ECG signals provide critical insights into the heart’s operational status, deploying efficient ECG electrodes is essential for routine monitoring (Figure 4b). However, the heart’s dynamic physiological environment, characterized by continuous expansion and contraction, presents significant challenges for ECG monitoring when using conventional soft materials.

Conductive hydrogels, particularly those with adhesive properties, offer notable advantages in this context [295]. These hydrogels exhibit excellent electrical conductivity, biocompatibility, and flexibility, allowing for stable adherence to skin or organ surfaces. Their ability to accommodate the heart’s rhythmic movements supports long-term, reliable ECG monitoring. Consequently, functional conductive hydrogels are regarded as optimal materials for precise and efficient cardiac monitoring, enabling improved diagnostic accuracy and patient outcomes.

#### 4.1.3. Other Applications

Conductive hydrogels are also highly effective for monitoring EMG signals from various organs, such as the bladder (Figure 4c) [296]. These hydrogels provide excellent mechanical adaptability, electrical conductivity, and bio-adhesiveness, ensuring stable attachment to the bladder surface while maintaining signal detection capabilities, even with bladder expansion of up to 200%. The hydrogel’s flexibility minimizes mechanical mismatch with the bladder, while its conductivity enhances signal transmission efficiency, reducing signal loss. In addition, injectable functional hydrogel composites offer a minimally invasive approach for electrode insertion into muscles, eliminating the need for large surgical incisions [34]. Compared to traditional electrode insertion methods, injectable conductive hydrogels require only small incisions, reducing the risk of scarring, pain, and infection. These features contribute to the high reliability and stability of EMG signal monitoring, enabling consistent and long-term signal recording [297].

### 4.2. Therapy 

Conductive hydrogels have a wide range of applications in disease treatment and tissue regeneration. Their exceptional conductivity and mechanical properties, which closely resemble those of biological tissues, help minimize mechanical mismatch and reduce physical damage when applied to organs. This capability significantly enhances therapeutic outcomes for conditions such as myocardial infarction and cardiac arrhythmias through electrical stimulation. In neural applications, conductive hydrogels effectively suppress neuroinflammation and promote neuronal regeneration. Additionally, their three-dimensional network structure is crucial for regulating the complex microenvironment needed for bone regeneration and for accelerating wound healing by enabling the controlled release of drugs and nanoparticles. This section provides an in-depth examination of the various therapeutic applications of conductive hydrogels, highlighting their versatility in enhancing clinical outcomes across different medical fields.

#### 4.2.1. Disease Treatment

The treatment of cardiovascular diseases, particularly myocardial infarction (MI), requires advanced materials capable of supporting cardiac function and repair [298,299]. Conductive hydrogels have emerged as a highly promising solution due to their unique combination of properties that align well with the needs of heart tissue.

These hydrogels possess a mechanical modulus similar to that of cardiac tissue, minimizing mechanical mismatch and reducing physical damage when applied to the heart’s surface (Figure 4d). This compatibility enhances the effectiveness of electrical stimulation and supports cellular activity, which is critical for treating cardiovascular conditions. By adhering securely to the heart’s surface, conductive hydrogels help mitigate common complications of MI, such as arrhythmias and asynchronous contractions of the cardiac muscle [300]. These issues often result from disruptions in electrical signal transmission within the heart muscle.

The ability of conductive hydrogels to reduce mechanical mismatch and physical damage at the interface with cardiac tissue improves the propagation of electrical signals, facilitating better transmission between myocardial cells. This supports synchronized cardiac contractions and helps restore blood flow. Furthermore, the electrical properties of conductive hydrogels can regulate irregular electrical signals in the atria, aiding in the management of arrhythmias such as ventricular tachycardia. Recent studies have demonstrated the effectiveness of injectable conductive hydrogel nanocomposites in treating MI in pig models, showcasing their potential as a transformative approach in cardiovascular therapy [301].

#### 4.2.2. Tissue Regeneration

Effective tissue regeneration requires materials capable of addressing the complex challenges associated with tissue damage and repair. Conductive hydrogels have emerged as a promising solution due to their unique combination of properties, including high electrical conductivity, mechanical flexibility, and biocompatibility (Figure 4e). These characteristics enable conductive hydrogels to interact seamlessly with biological tissues, facilitating various regenerative processes.

In the context of traumatic brain injury, conductive hydrogels offer significant advantages. They adhere strongly to brain tissue, providing soft, supportive interfaces that help mitigate mechanical damage and reduce inflammation [302]. By mimicking the mechanical properties of brain tissue, these hydrogels delay swelling and atrophy, while also suppressing neuroinflammation and promoting neuronal survival. Additionally, the electrical stimulation capabilities of conductive hydrogels aid neuronal recovery and functional restoration by mimicking neurotransmitter activity and forming conductive bridges between neurons, which promotes neural growth and regeneration.

Beyond brain injury, conductive hydrogels are also highly effective in the regeneration of peripheral nerves and spinal cord injuries. Their directional structure supports the alignment and growth of nerve cells, enhancing cell proliferation through electrical stimulation [303,304]. This alignment fosters directional axonal growth and facilitates the exchange of metabolic substances, both of which are crucial for nerve cell recovery and functional restoration. The electrical stimulation provided by these hydrogels further enhances nerve cell proliferation and growth, making them highly valuable for treating nerve injuries.

In bone treatment and regeneration, conductive hydrogels play a critical role by regulating the complex bone microenvironment, which involves various cell types (e.g., osteoblasts, osteocytes, immune cells) and matrix components, as well as numerous growth and signaling factors within the extracellular matrix (ECM) [305]. Conductive hydrogels can be tailored to provide structural support, activate bone formation pathways, and modulate cellular behaviors, thereby promoting efficient bone regeneration.

Overall, the versatile properties of conductive hydrogels enable them to address a wide range of challenges in tissue regeneration, making them indispensable in advancing treatments for brain, nerve, and bone injuries.

#### 4.2.3. Wound Healing

Conductive hydrogels are increasingly recognized for their pivotal role in wound healing due to their unique structural and functional properties. Their three-dimensional network and high moisture content closely mimic the ECM, enhancing their ability to interact effectively with cells and biological molecules. This similarity enables conductive hydrogels to serve as excellent platforms for the delivery and encapsulation of bioactive molecules, such as growth factors, DNA, and small interfering RNA [306].

The inherent electrical and structural properties of conductive hydrogels facilitate the application of electrical stimuli, which induces both chemical and physical changes within the hydrogel (Figure 4f). These changes play a key role in regulating the encapsulation and release of drugs and nanoparticles, neutralizing reactive oxygen species, and promoting oxygen production. Through these mechanisms, electrical stimulation provided by conductive hydrogels accelerates the wound healing process.

Electrical stimulation also allows for controlled release of therapeutics from the hydrogel, ensuring targeted delivery of essential substances to the wound site. This targeted approach reduces inflammation and promotes a more efficient healing response, leading to faster wound recovery. Therefore, conductive hydrogels not only support the physical repair of wounds but also enhance the biochemical environment necessary for optimal healing.

## 5. Applications of Hydrogels in the Biological and Therapeutic Domain

This section highlights the diverse applications of conductive hydrogels in monitoring biological signals, providing electrical stimulation, and facilitating therapeutic interventions across various organ systems. Specifically, we examine their roles in the brain, peripheral nervous system (PNS), and heart, focusing on how these hydrogels contribute to precise signal monitoring and targeted treatments.

### 5.1. Applications of Conductive Hydrogels and Nanocomposites in Neural Signal Recording

The brain is an exceptionally complex biological system that requires efficient conduction of nerve signals and precise neural stimulation. Its extensive neural networks and intricate neuronal arrangements are essential for accurate signal transmission and modulation. Therefore, conductive hydrogels and hydrogel nanocomposites used for neural interfaces must exhibit high electrical conductivity to efficiently record and transmit low-intensity nerve signals. Additionally, they must possess high biocompatibility to minimize inflammatory responses and ensure long-term stability. Furthermore, these hydrogels should have mechanical properties similar to the soft brain tissues to adapt effectively to the micro-movements and deformations of neural tissues.

**Figure 5:**
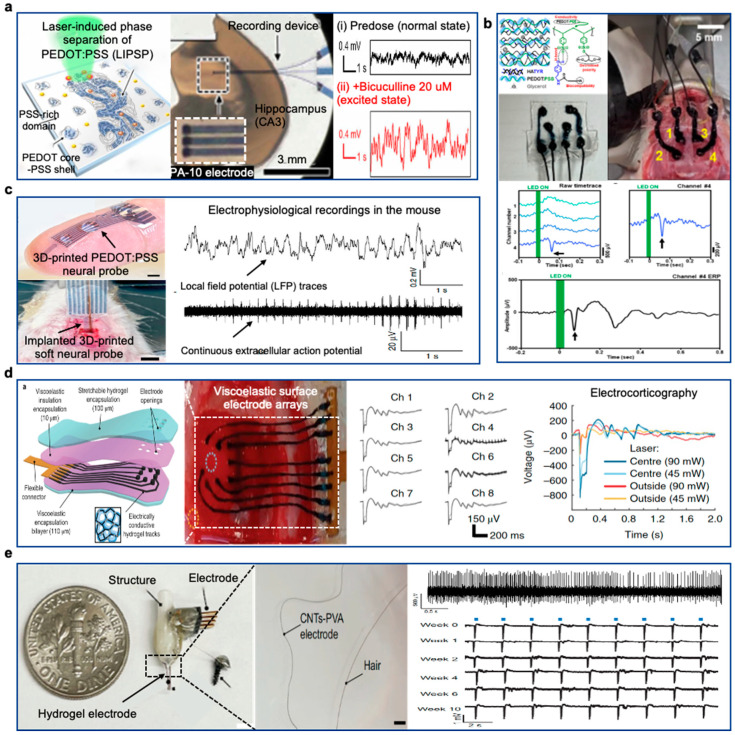
Conductive hydrogels and nanocomposites in soft implantable electronics for brain neural recording. (**a**) PEDOT:PSS conductive hydrogel electrode fabricated by laser-induced phase separation. Reproduced with permission from Ref. [32], Copyright 2022, AAAS. (**b**) Injectable and tissue-conformable conductive hydrogel for MRI-compatible brain electrodes. Reproduced with permission from Ref. [307], Copyright 2023, OAE Publishing, Inc. (**c**) 3D printable conducting polymer for soft neural probes. Reproduced with permission from Ref. [308], Copyright 2020, Springer Nature. (**d**) Fully viscoelastic electrode for stimulation and recording of electrocorticograms on a rat cortical surface. Reproduced with permission from Ref. [309], Copyright 2021, Springer Nature. (**e**) Hydrogel optoelectronic device (optrode) for electrophysiology recording signals from the mouse VTA. Reproduced with permission from Ref. [310], Copyright 2024, Springer Nature.

For brain signal monitoring, PEDOT:PSS is a widely used material due to its extraordinary electrical, mechanical, and swelling properties. Won et al. developed a conductive hydrogel electrode using a PEDOT:PSS and gold nanoparticles (AuNPs) nanocomposite ink. Typically, PEDOT:PSS has limitations due to its structure, where PEDOT forms the conductive core and PSS forms an insulating shell, leading to instability and fragmentation in moist environments. To address this, Won et al. redesigned the phase distribution of PEDOT:PSS through a laser-induced phase separation method (Figure 5a) [32]. This technique involved laser-induced processing to separate and recrystallize PEDOT and PSS, enhancing the hydrogel’s electrical conductivity and water stability.

The resulting hydrogel exhibited a maximum electrical conductivity of ~670 S/cm in deionized water and demonstrated durability, maintaining performance even after multiple swelling and drying cycles. The hydrogel was patterned as a neural recording electrode (referred to as PA-10 electrode) with a diameter as small as approximately 6 μm. The electrode maintained stable electrochemical properties over six months in physiological environments, demonstrating long-term stability in bioelectronic devices. It was also used to record in situ local field potential (LFP), which measures the electrical field potential in the brain’s extracellular space. The electrode’s excellent electrical properties and fine spatial resolution allowed for stable spatiotemporal recording of LFP signals, capturing variations depending on the state of the mouse brain.

Kim et al. developed an innovative injectable conductive hydrogel (ICH) using tyramine-conjugated hyaluronic acid (HATYR) and PEDOT:PSS for Magnetic Resonance Imaging (MRI)-compatible brain-interfacing electrodes (Figure 5b) [307]. The ICH demonstrated low impedance of approximately 5 kΩ at 10 Hz, indicative of its high volumetric capacitance. Due to the biocompatibility of HATYR, histological and cytotoxicity assays revealed minimal inflammation and toxicity. Additionally, the ICH degraded to approximately 40% of its original volume within four weeks in vivo.

The ICH is injectable and can be easily patterned via syringe onto a stretchable and flexible elastomeric substrate, allowing it to conform to curvilinear brain tissues and effectively record ECoG signals under light stimulation. Notably, MRI imaging of the implanted devices showed no artifacts, highlighting the potential of these hydrogel electrodes for advanced ECoG applications.

The ICH offers several advantages for implantable bioelectronics, including the ability to form high-resolution linear arrays (less than 200 μm in diameter) due to ionic crosslinking between the tyramine and sulfonate groups. It also exhibits high biocompatibility, with an in vitro cell viability rate of approximately 95.9% and no signs of inflammation or fibrosis in vivo. Furthermore, the ICH effectively recorded visual evoked potentials from the visual cortex in a rodent model, showcasing its potential for neural signal monitoring and other advanced applications in neuroelectronics.

PEDOT:PSS can be effectively utilized in neural probes through 3D printing technology. Zhao et al. introduced a high-performance 3D-printable conducting polymer ink based on PEDOT:PSS for creating intricate 3D structures (Figure 5c) [308]. To prepare the ink, they first freeze the aqueous PEDOT:PSS solution and lyophilize it to isolate the PEDOT:PSS nanofibrils. These nanofibrils are then re-dispersed in a mixture of water and DMSO to produce a high-concentration ink suitable for 3D printing. This ink enables the printing of structures with high resolution (over 30 μm) and significant height (more than 20 layers). The printed structures are subsequently dry-annealed to improve their conductivity and flexibility, and then swollen in water to convert them into a soft PEDOT:PSS hydrogel, maintaining a conductivity of up to 28 S/cm. The 3D-printed PEDOT:PSS was used to fabricate a soft neural probe for in vivo bioelectronic signal recording. This probe, which can be produced in less than 20 min, combines both insulating and conductive materials and features nine PEDOT:PSS electrode channels with a diameter of 30 μm. After assembly, the probe was successfully implanted into the hippocampus of a mouse, where it recorded continuous neural activity from freely moving subjects, including local field potentials. This demonstrates the potential of 3D-printed PEDOT:PSS for developing advanced neural probes with precise structural control and excellent bioelectronic performance.

For effective brain signal recording, addressing the mechanical mismatch between rigid implantable electrode arrays and soft biological tissues is crucial. Traditional rigid electrodes can cause tissue damage due to this mismatch and their lack of viscoelastic properties, while biological tissues are viscoelastic and deform permanently under stress. To overcome these limitations, Mooney et al. developed a novel electrode array using highly porous viscoelastic hydrogels embedded with conductive carbon nanomaterials (Figure 5d) [309]. This approach allows the electrodes to conform to the complex geometry of soft tissues, reduces mechanical mismatch, and maintains electrical conductivity, enhancing both mechanical and electrical performance.

To create these highly porous viscoelastic hydrogels, Mooney et al. began with alginate hydrogels and used various cross-linking agents to fine-tune their viscoelastic and mechanical properties. They analyzed the mesh size and surface tension of these hydrogels and fabricated substrates using polyimide, Ecoflex, and alginate. For the electrically conductive gel-based interconnects, they incorporated pyrene-modified graphene flakes (GFs) and CNTs into the soft alginate hydrogels to enhance ionic conductivity. By freezing the gels before cross-linking, they created microporous structures that reduced the percolation threshold of the conductive particles.

Both nanoporous and microporous conductive gels were cast into molds, allowing them to adapt well to complex tissue geometries and withstand significant bending and knotting without damage. The microporous conductive gels (microCGs) achieved conductivity values exceeding 10 S/m with less than 2% carbon loading, with CNTs proving more effective than GFs in enhancing conductivity.

Mooney et al. successfully used these electrode arrays to record neural activity from the dura of a Thy1 rat. By directing a laser at the center of the array, they induced electrical activity in the cortex and observed variations in neural activity based on different laser positions and power levels. The recorded signals were confirmed to originate from underlying neurons, with the amplitude of the recorded activity comparable to that of existing electrode arrays of similar diameter. This demonstrated the potential of using viscoelastic conductive hydrogels to improve brain signal recording while minimizing tissue damage.

On the other hand, CNTs have also been fabricated into fiber forms for neural signal measurement. Huang et al. developed a conductive hydrogel interface, termed an optrode, based on CNTs and PVA, to demonstrate electrical stimulation in the brain (Figure 5e) [310]. The optrode exhibited excellent impedance characteristics (~658 ± 277 kΩ at 1 kHz), high stretchability (approximately 139.3–169.2%), and a low elastic modulus (~2.8–9.3 MPa). It maintained stable structural performance, with diameter changes within 1% over more than three months in a buffer that mimicked physiological conditions (37 °C, pH 6–8). When implanted in the mouse ventral tegmental area, the optrode enabled simultaneous optogenetic stimulation and electrophysiological recording, facilitating real-time monitoring and analysis of brain neural activity. The optrode successfully recorded spontaneous neural spikes with high signal-to-noise ratios (SNR), providing clear differentiation of neural activity. Its flexible nature minimized tissue damage while maintaining long-term stable recording performance. This study demonstrated the potential of CNT-based optrodes for neural stimulation and recording with reduced tissue disruption and high precision.

### 5.2. Applications of Conductive Hydrogels and Nanocomposites for ECG Monitoring and MI Therapy 

The heart operates through a continuous cycle of contraction and relaxation, driven by rapid electrical signal conduction. This dynamic process places substantial mechanical stress on cardiac tissues, underscoring the need for materials that can accommodate such stresses while maintaining effective functionality. Conductive hydrogels and hydrogel nanocomposites, with their inherent flexibility and electrical conductivity, are particularly well-suited for applications involving ECG monitoring and cardiac stimulation. These hydrogels offer a valuable solution for integrating with cardiac tissues due to their ability to conform to the heart’s surface and endure mechanical movements.

The flexibility of these hydrogels ensures they can withstand the continuous expansion and contraction of the heart, minimizing the risk of mechanical mismatch and damage. Additionally, their high electrical conductivity enhances the accuracy of signal recording and stimulation, making them ideal for both diagnostic and therapeutic applications in cardiology.

Recent research has focused on optimizing the performance of conductive hydrogels for cardiac use. Studies are exploring various formulations and designs to improve their integration with heart tissues, enhance their durability under mechanical stress, and refine their electrical properties for better performance. These advancements aim to leverage the potential of conductive hydrogels to improve ECG monitoring, provide targeted cardiac stimulation, and support overall cardiac health.

**Figure 6:**
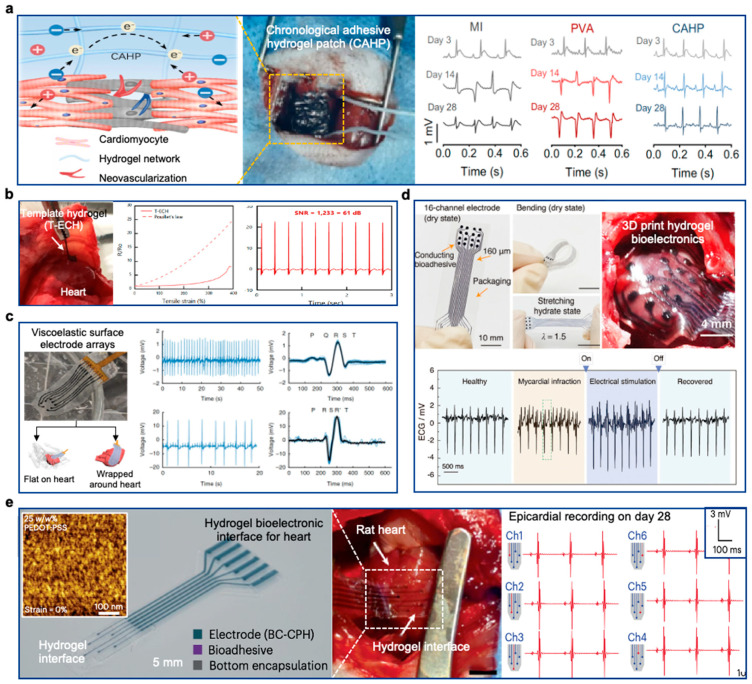
Conductive hydrogels and nanocomposites in soft implantable electronics for ECG monitoring and MI therapy. (**a**) Chronological adhesive hydrogel patch for synchronous mechanophysiological monitoring and electrocoupling therapy. Reproduced with permission from Ref. [300], Copyright 2023, Springer Nature. (**b**) Highly conductive tissue-like hydrogel interface based on template-directed in-situ synthesis. Reproduced with permission from Ref. [311], Copyright 2023, Springer Nature. (**c**) Fully viscoelastic electrode on a mouse heart, showing the recorded ECG with filtered and averaged beats. Reproduced with permission from Ref. [309], Copyright 2021, Springer Nature. (**d**) 3D-printed hydrogel bioelectronics for electrophysiological monitoring and electrical modulation. Reproduced with permission from Ref. [312], Copyright 2023, John Wiley and Sons. (**e**) 3D-printable high-performance conducting polymer hydrogel for all-hydrogel bioelectronic interfaces. Reproduced with permission from Ref. [30], Copyright 2023, Springer Nature.

Yu et al. introduced a chronological adhesive hydrogel patch (CAHP) composed of functionalized PANIS (f-PANI) and PVA, designed for high tissue adhesion, rapid self-healing capabilities, and superior electrochemical properties (Figure 6a) [300]. The f-PANI was synthesized using a side-chain modification strategy to enhance its conductivity and hydrophilicity in physiological media. This was achieved through the oxidative polymerization of aniline, 3-aminobenzeneboronic acid, and 3-aminobenzoic acid, which incorporated borate and carboxyl side chains into f-PANI, forming borate ester bonds and hydrogen bonds, respectively. These modifications improved the conductivity and physical and chemical stability of f-PANI, resulting in excellent conductivity (~1.35 S/m) and stable dispersion in water.

The synthesized f-PANi was mixed with PVA to create a viscous, flowable gel, which was used to fabricate the CAHP. The hydrogel could be injected and shaped into patches using a custom applicator, allowing for easy application onto the heart’s surface during minimally invasive surgery. The CAHP exhibited shear-thinning properties, meaning its viscosity decreased with increasing shear rate to prevent blockage during injection. Importantly, the viscosity recovered to its original level after the shear was removed, demonstrating that the dynamic cross-linking effectively maintained the mechanical properties of the hydrogel.

To verify the CAHP’s simultaneous therapeutic effect on MI, ECG recordings were analyzed to assess its impact on cardiac electrophysiology. The CAHP alleviated ST-segment elevation, shortened the QRS interval, and improved overall cardiac electrophysiological behavior. These results demonstrated that the CAHP effectively compensated for electrical conduction deficits, activated cardiomyocyte action potentials, and alleviated the electrical decoupling caused by MI.

Similar to previous research, achieving high conductivity, softness, stretchability, and strong adhesion to biological tissues is crucial for long-term stability in conductive hydrogels. To meet these requirements, Kang et al. developed a novel template-directed assembly method (Figure 6b) [311]. The process began with the preparation of a polyacrylic acid (PAA) template. PEDOT:PSS and acrylic acid (AA) monomer solutions were mixed to create the initial blend. The PAA template network was formed through the polymerization of AA monomers using a crosslinker. DMSO was then added to transform PEDOT:PSS from a colloidal state into extended nanofibers, which aligned along the template network. Finally, the solvents were removed through drying, and the material was re-swollen in water, resulting in a hydrogel with high electrical conductivity and mechanical strength.

The resulting hydrogel, termed T-ECH, exhibited properties similar to cardiac tissue, with elasticity (~25 kPa), high conductivity (~247 S/cm), stretchability (~610%), toughness (~1 MJ/m^3^), and high water content (~90 wt%). Additionally, T-ECH demonstrated strong adhesion to wet tissues, making it highly suitable for biomedical applications. T-ECH electrodes performed exceptionally well in ECG monitoring, maintaining stable contact with the wet, dynamic environment of a continuously beating heart without requiring additional fixatives. Thanks to its high conductivity, the T-ECH electrodes achieved stable and high-quality ECG recordings, with an outstanding SNR of 1009 (60.08 dB), surpassing the performance of other ECG devices.

Additionally, the highly porous, viscoelastic hydrogel electrode array developed by Mooney et al., as described earlier in Figure 5d, effectively recorded cardiac electrical signals when inserted into the heart (Figure 6c) [309]. This viscoelastic surface electrode array seamlessly integrated with the heart tissue. The array was designed with eight electrodes arranged in a 3 × 3 grid, with each electrode having a diameter of 700 μm and spaced 800 μm apart, leaving a central void. The array remained flat on the mouse heart due to surface tension and plastic deformation, allowing it to record ECG signals with a maximum SNR of 17.4. Even when wrapped around the heart and bent over 180°, the electrodes maintained functionality and recorded ECG signals with an SNR of 15.6, demonstrating their flexibility and durability in cardiac applications.

Multi-signal mapping is essential for diagnosing and monitoring complex cardiac conditions. To address this need, Liu et al. developed a high-performance 3D-printable hydrogel based on PEDOT:PSS using direct ink writing technology (Figure 6d) [312]. The hydrogel precursor ink, composed of PEDOT:PSS, PVA, chitosan, and poly(acrylic acid-co-acrylic acid N-hydroxysuccinimide ester) (PAA-NHS), was printed into complex 2D and 3D structures. The ink exhibited favorable rheological properties, allowing for smooth printing and rapid recovery after extrusion. Following the printing process, the structures were air-dried, leading to chemical cross-linking and hydrogen bonding that enhanced mechanical strength. The resulting hydrogel, with a Young’s modulus of ~650 kPa, closely matched the mechanical properties of cardiac tissues, minimizing mechanical mismatches. It also demonstrated robust bio-adhesion with an interfacial toughness of ~200 J/m^2^ and a shear strength of ~120 kPa, ensuring stable attachment.

The hydrogel offered long-term structural and electrochemical stability with a conductivity of over ~9 S/m. Using the 3D-printed hydrogel, they fabricated a multi-layered 3D-printed hydrogel bioelectronic device with a 4 × 4 electrode array, forming a thin film (120 µm thick). This bioelectronic device was implanted on the surface of the heart, where it accurately recorded and analyzed electrical signals from the rat’s heart, detecting abnormal ST-segment elevation in a chemically induced acute myocardial infarction model. The hydrogel electrodes adhered well to the heart, recorded signals in sync with the dynamic heartbeat, and were able to restore heart rhythm through electrical stimulation.

Similarly, Zhou et al. employed the 3D printing method to develop a dual-continuous conductive polymer hydrogel (BC-CPH) that meets the demands for high electrical conductivity and mechanical strength (Figure 6e) [30]. The BC-CPH was created using PEDOT:PSS for the electrical phase and highly stretchable hydrophilic polyurethane for the mechanical phase, dissolved in a solvent mixture of ethanol and water. After the solvent evaporated at room temperature and the material equilibrated in physiological conditions, a stable bi-continuous structure was formed, incorporating both mechanical and electrical phases. As a result, the hydrogel exhibited over 200% recoverable strain and a high fracture toughness exceeding 3000 J/m^2^. The BC-CPH was further adapted for 3D printing into bioelectric interfaces with six channels, which were used for electrophysiological recording and stimulation experiments in the heart. The experiments demonstrated the long-term stability and reliable biointerface capabilities of the BC-CPH, making it an effective tool for cardiac monitoring and therapy.

### 5.3. Applications of Conductive Hydrogels and Nanocomposites in Neural Signal Recording and Stimulation

The nervous system, including both PNS and central nervous system (CNS), is crucial for connecting various body parts with the brain and spinal cord, thereby regulating sensory and motor functions. Effective monitoring and treatment of nerve damage and neurological disorders are essential for maintaining these critical functions. Conductive hydrogels and hydrogel nanocomposites are highly beneficial for these purposes due to their high electrical conductivity, enabling precise recording and transmission of nerve signals, and their excellent biocompatibility, which minimizes adverse reactions with biological tissues. Additionally, the mechanical flexibility of conductive hydrogels allows them to conform to and integrate smoothly with nerve tissues, accommodating the dynamic nature of neural structures in both the PNS and CNS. This flexibility helps reduce mechanical mismatch and long-term damage, making these materials ideal for both monitoring and therapeutic interventions in the treatment of nerve injuries and neurological disorders.

**Figure 7:**
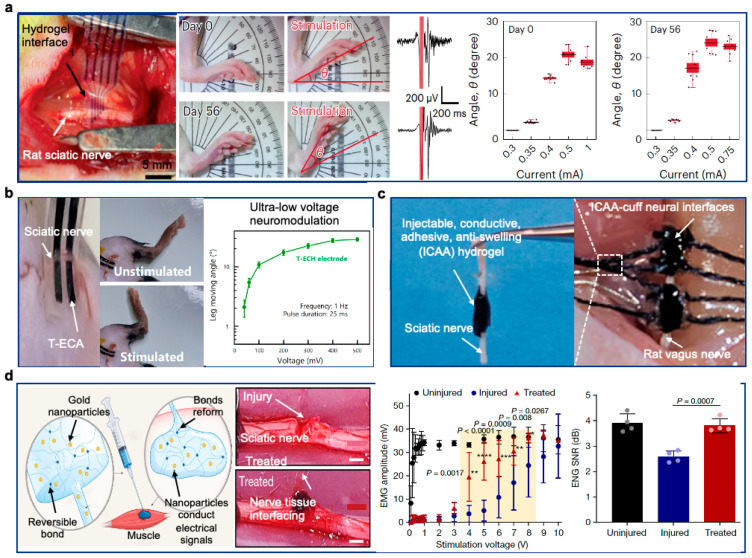
Conductive hydrogels and nanocomposites in soft implantable electronics for in vivo neural signal recording and stimulation in the nervous system. (**a**) A 3D-printable high-performance conducting polymer hydrogel for all-hydrogel bioelectronic interfaces. Reproduced with permission from Ref. [301], Copyright 2023, Springer Nature. (**b**) Highly conductive tissue-like hydrogel interface through template-directed assembly. Reproduced with permission from Ref. [293], Copyright 2023, Springer Nature. (**c**) Highly stable, injectable, conductive hydrogel for chronic neuromodulation. Reproduced with permission from Ref. [313], Copyright 2024, Springer Nature. (**d**) Injectable IT-IC hydrogel interfacing for instantaneous closed-loop rehabilitation. Reproduced with permission from Ref. [300], Copyright 2023, Springer Nature. *p*-values are indicated with asterisks: * *p* < 0.05, ** *p* < 0.01, *** *p* < 0.001, **** *p* < 0.0001.

Fine peripheral nerves, less than 300 μm in diameter, have unique anatomical variations and are mechanically fragile, requiring materials that can adhere well to soft and small surfaces. To meet these requirements, Zhou et al. developed 3D-printable hydrogel bioelectronics, which were used to effectively stimulate the sciatic nerves in rats and successfully stimulate the hindlimbs (Figure 7a). The tissue-adhesive nature of these hydrogel bioelectronics enabled effective signal recording and transmission. As a result, similar to their use in cardiac signal recording (Figure 5e) [301], this device provided stable and long-term electrophysiological recording and stimulation of the sciatic nerves. Notably, a significantly greater hindlimb joint angle movement was observed on day 56 compared to day 0 post-implantation during sciatic nerve stimulation, highlighting the potential for long-term functional recovery using these hydrogel bioelectronics.

Kang et al. developed the T-ECH hydrogel, which features tissue-like elasticity, high tissue adhesion, high conductivity, and high stretchability (Figure 5b) [293]. They demonstrated the potential for nerve modulation by implanting these electrodes in the sciatic nerve (Figure 7b). The T-ECH electrodes successfully stimulated the sciatic nerve and maintained stable electrical interfacing despite continuous leg movement, thanks to their mechanical properties and strong adhesion to biological tissue. The electrodes enabled ultra-low-voltage nerve modulation, achieving electrically induced leg movements at a stimulation voltage of only 40 mV without penetrating the nerve or removing the epineurium.

Additionally, injectable hydrogels play a significant role in nerve modulation by filling the space between the cuff electrode and the nerve, adapting to various shapes and sizes while reducing mechanical stress and improving overall fit and comfort. Extensive research has explored injectable conductive hydrogels and hydrogel nanocomposites for these purposes. Luo et al. introduced tannic acid to modulate the gelation rate and enhance the injectability of an injectable, conductive, adhesive, and anti-swelling hydrogel (ICAA hydrogel) (Figure 7c) [313]. The gelation time and mechanical properties were optimized by adjusting the solid content, and the effects of environmental factors such as temperature, pH, and ionic strength were evaluated. As a result, the optimized ICAA hydrogel exhibited a Young’s modulus ranging from ~6.42 kPa to 40.9 kPa, minimal mass loss and volume change after 4 weeks in PBS at 37 °C, high conductivity (~92.43 ± 7.65 S/m), and strong adhesion to tissue (~20.9 kPa). The ICAA hydrogel was successfully used to create a neural interface for the vagus nerve.

Jin et al. developed an injectable tissue-interfacing conductive hydrogel (IT-IC hydrogel) for peripheral nerve stimulation using a modified Suzuki-Miyaura reaction (Figure 7d) [300]. The IT-IC hydrogel was created through a multi-crosslinking strategy with phenylborate (PB), resulting in a complex structure with irreversible biphenyl bonds, reversible coordinate bonds, and weak multivalent ionic interactions. The interaction between AuNPs and PB groups enhanced the hydrogel’s conductivity, while hydrogen bonds, metal-π bonds, and metal-carboxylate bonds improved its physical properties. The hydrogel demonstrated high electrical conductivity (~10⁻^2^ S/cm) and exhibited exceptional mechanical and electrical performance, including effective energy dissipation and recovery during injection. It maintained stable electrical resistance and conductivity under 100% deformation, with minimal electrical hysteresis and consistent impedance during mechanical deformation.

The IT-IC hydrogel also exhibited high tensile adhesion strength (~8.8 ± 2.1 kPa) to nerve tissues due to its stretchability, modulus matching, low impedance, and pattern-controllable injectability. When injected into the rat sciatic nerve, the hydrogel’s adhesion remained stable, even under stretching or bending conditions, without delamination. The IT-IC hydrogel effectively recorded various sensory neural responses at the nerve interface, with SNR for different mechanical stimulations (strong brushing, weak brushing, and tapping) comparable to previously reported sensory neural signals. Additionally, nerve stimulation at various frequencies and amplitudes successfully induced muscle contractions without tissue damage, demonstrating precise control over the nerve-to-muscle signaling pathway. The trend of ankle contractions in response to applied voltages matched EMG signals, further confirming the hydrogel’s capability for accurate nerve modulation. The brief summary of the hydrogel applications introduced in this text can be found in Table 1.

## 6. Current Limitations and Prospects

Although several notable results about conductive hydrogel and hydrogel nanocomposites for implantable biomedical electronics have been reported recently, there is still room for elaboration of their applicability as bioelectronics. In this section, we will summarize the advantages of soft hydrogel bioelectronics compared to rigid electronics, and discuss current limitations of conductive hydrogels and hydrogel nanocomposites and future perspectives.

### 6.1. Advantages of Soft Conductive Hydrogels for Implantable Bioelectronics

Conductive hydrogels have low modulus and high stretchability, making them mechanically compatible with soft tissues like the heart, brain, and muscles. This allows them to conform to the tissue surface, reducing mechanical mismatch and minimizing irritation or injury at the tissue-device interface. Due to their softness and stretchability, conductive hydrogels exert less pressure on surrounding tissues, reducing the risk of chronic damage or necrosis. Long-term implants made from hydrogels are less likely to induce pressure sores, ulcers, or necrosis compared to rigid materials. In contrast, rigid materials like metal and silicon can cause chronic inflammation or scarring due to their stiffness, which differs greatly from the soft, elastic nature of biological tissues. Not only mechanically soft, most of these hydrogels also exhibit chemical biocompatibility. Their hydrophilic nature allows better integration with the body’s aqueous environment, reducing the risk of immune responses or fibrous encapsulation that can impair the device’s performance. Rigid materials like metals and silicon are prone to induce a foreign body response, which can lead to tissue encapsulation and degrade electrical signal quality over time.

Conductive hydrogels can conform and adhere tightly to complex, curved tissue surfaces, maintaining intimate contact and improving signal transmission for both sensing and stimulation applications. Their flexibility allows them to accommodate dynamic movement, such as the constant beating of the heart or brain pulsations, without causing tissue damage. Rigid materials do not offer this flexibility, which can lead to detachment or gaps forming between the implant and tissue, reducing the device’s effectiveness. Conductive hydrogels can be engineered to have tunable mechanical, electrical, and adhesive properties by modifying their polymer structure or incorporating different nanomaterials. This flexibility allows them to be customized for specific biomedical applications (e.g., neural implants, cardiac patches, or drug delivery systems).

### 6.2. Current Obstacles and Endeavors

Ensuring that conductive hydrogels are biocompatible and do not induce adverse immune responses presents a significant challenge. While many base polymers are inherently biocompatible, the incorporation of conductive elements, such as certain conductive polymers or nanofillers, can introduce potential toxicity concerns. Achieving the right balance between biodegradability and stability is equally challenging, as materials must degrade at a controlled rate without causing harm or losing functionality prematurely. To address these issues, the development of biocompatible and biodegradable conductive materials—such as bio-derived conductive polymers and safe nanomaterials—is crucial. Advances in polymer chemistry and surface modification techniques will also play a key role in creating safer, more effective materials.

Another challenge lies in the mechanical durability of conductive hydrogels, which must withstand the physical stresses of the body while maintaining flexibility and softness. Although these hydrogels offer remarkable stretchability, their long-term mechanical performance under continuous deformation—such as heartbeats or muscle movements—can be lower than that of traditional rigid materials. Over time, hydrogels may degrade, lose adhesion, or experience fatigue, reducing their lifespan in chronic applications. The incorporation of conductive elements often compromises mechanical properties, making hydrogels more brittle. To enhance durability and robustness, researchers are focusing on engineering hydrogels with tunable mechanical properties through strategies like crosslinking density control, hybrid composites, and toughening agents.

In addition to mechanical challenges, the hydrophilic nature of conductive hydrogels poses its own difficulties. Their ability to absorb water can lead to swelling, which alters their physical and electrical properties over time. This can impair the function of implanted devices. Conversely, dehydration of the hydrogel may cause a loss of flexibility and conductivity, further diminishing their effectiveness. In contrast, rigid materials do not experience these fluctuations, as their properties remain stable regardless of environmental changes.

Maintaining stable electrical conductivity in the body’s physiological conditions is another obstacle. The presence of ions, proteins, and other biological molecules can interfere with conductive pathways, leading to degradation or fouling of the hydrogel’s components. Developing stable doping strategies, protective coatings, and antifouling surfaces will be critical for ensuring long-term conductivity. Additionally, advances in nanocomposite design, especially the incorporation of stable and conductive nanomaterials, will be essential for improving the electrical stability of these materials over time.

While conductive hydrogels achieve good conductivity by incorporating materials like gold or silver nanowires, their conductivity still falls short of pure metals like gold, platinum, or silicon. This limits their performance in applications requiring high-precision electrical signals or long-term stability in sensing and stimulation. Furthermore, fabricating conductive hydrogels with uniform conductivity and mechanical properties is more complex and expensive compared to traditional materials. The scalability of producing high-quality hydrogels and nanocomposites with consistent performance remains a challenge. The precise control needed over nanomaterial dispersion within the hydrogel matrix adds further complexity. Emerging technologies such as 3D printing and microfabrication offer promising solutions by allowing fine control over material properties and architecture. However, scalable production methods that ensure uniformity and reproducibility will be crucial for clinical translation.

Moreover, the integration of conductive hydrogels with traditional rigid electronic components, which are typically made of metals and semiconductors, presents additional challenges. The soft, stretchable nature of hydrogels can complicate the establishment of stable electrical connections with rigid components, potentially increasing fabrication complexity and costs.

### 6.3. Prospects for Next-Generation Biomedical Conductive Hydrogels

The development of smart conductive hydrogels that can respond to environmental stimuli (e.g., pH, temperature, light, electric fields) offers exciting possibilities for on-demand drug delivery, dynamic tissue scaffolds, and soft robotics. These systems could adapt to changing conditions in the body, providing tailored therapeutic responses. Future conductive hydrogels and nanocomposites may incorporate multiple functionalities, such as simultaneous sensing and actuation, drug delivery, and electrical stimulation. This multifunctionality could revolutionize implantable devices, enabling more comprehensive monitoring and treatment. Advances in material design and manufacturing technologies could enable the customization of conductive hydrogels for individual patients, taking into account their specific anatomical and physiological conditions. This personalization could improve the efficacy and safety of medical devices.

A major challenge facing all implantable electronics is developing effective strategies for power and data transfer. Recently, technologies such as in situ energy harvesting, implantable batteries, and wireless power transfer have emerged as potential solutions for powering soft implantable devices. However, the practical implementation of these techniques is challenging due to several factors. The relatively low conductivity of conductive hydrogels, their water permeability, and the difficulty of establishing reliable interconnections with conventional integrated circuit devices present significant obstacles. Currently, most data transmission relies on wired communication with external data acquisition systems. Looking ahead, advancements in wireless communication technologies, combined with enhanced conductivity and innovative wiring strategies, could enable fully wireless data transfer for implantable electronics. This shift would greatly improve their functionality and ease of use, paving the way for more versatile and user-friendly implantable devices.

The integration of conductive hydrogels with emerging technologies, such as bioelectronics, bioprinting, and AI-based analysis, could lead to new therapeutic approaches and diagnostic tools. The combination of electronic and biological functionalities could open new frontiers in medical treatment and monitoring. Conductive hydrogels and nanocomposites hold promise for regenerative medicine, particularly in the regeneration of electrically active tissues like nerves, muscles, and the heart. Advances in this field could lead to new treatments for conditions such as spinal cord injuries, heart disease, and neurodegenerative disorders. The push towards sustainable and environmentally friendly materials will likely influence the development of conductive hydrogels. The use of renewable resources, green synthesis methods, and recyclable materials will become increasingly important.

In conclusion, while there are significant challenges in the development and application of implantable conductive hydrogels and conductive hydrogel nanocomposites, the future holds immense potential. Continued research and innovation in material science, bioengineering, and nanotechnology will be key to overcoming current obstacles and unlocking new possibilities for these advanced materials in biomedical applications.

## Figures and Tables

**Table 1:** Summary of the materials and applications of conductive hydrogel and hydrogel nanocomposites as bioelectronics.

Hydrogel Matrix	Conductive Filler	Mechanical Modulus	Conductivity	Functionality	Application	Disease Model	Chronic Application	Ref.
PEDOT:PSS	Au NP	57 MPa	670 S/cm		Monitoring, stimulation	Brain, nerve	4 weeks	[32]
HA, Tyramine	PEDOT:PSS	0.2 MPa	0.0179 S/cm	Biodegradable	Monitoring	Brain	4 Weeks	[307]
PEDOS:PSS		1.1 MPa	155 S/cm		Monitoring	Brain	2 weeks	[308]
Aiginate	Graphene, CNT	1 MPa	35 S/m	Self-healing	Monitoring	Brain, muscle, heart		[309]
PVA	CNT	2.8 MPa		Injectable	Monitoring, optogenetics	Brain	10 weeks	[310]
PVA	PANI		1.35 S/m	Adhesive, self-healing, injectable	Monitoring, regeneration	Heart	4 weeks	[300]
PAA	PEDOT:PSS	25 kPa	247 S/cm	Adhesive	Monitoring, stimulation	Heart, muscle	2 weeks	[311]
PEDOT:PSS		650 kPa	9 S/m	Adhesive	Monitoring, stimulation	Heart	2 weeks	[312]
HPU	PEDOT:PSS	1 MPa	11 S/cm	Adhesive	Monitoring, stimulation	Heart, nerve	8 weeks	[30]
Gelatin	PPy		0.00052 S/cm	Biodegradable	Regeneration	Heart	4 weeks	[301]
PVA	CNT	2.8 Mpa	670 S/cm		Optogenetics		3 month	[302]
HA, PEDOT	PEDOT		5 kOhm		Monitoring	Brain	4 weeks	[303]
PEDOT	PEDOT	1 MPa	11 S/cm	Adhesive	Monitoring	Heart	2 months	[304]
Phenylborate	Au NP		0.01 S/cm	injectable	Regeneration, Monitoring	Muscle, Nerve	4 weeks	[305]
